# Atlantic water influx and sea-ice cover drive taxonomic and functional shifts in Arctic marine bacterial communities

**DOI:** 10.1038/s41396-023-01461-6

**Published:** 2023-07-08

**Authors:** Taylor Priest, Wilken-Jon von Appen, Ellen Oldenburg, Ovidiu Popa, Sinhué Torres-Valdés, Christina Bienhold, Katja Metfies, William Boulton, Thomas Mock, Bernhard M. Fuchs, Rudolf Amann, Antje Boetius, Matthias Wietz

**Affiliations:** 1 Max Planck Institute for Marine Microbiology, Bremen, 28359 Germany; 2 Physical Oceanography of the Polar Seas, Alfred Wegener Institute Helmholtz Centre for Polar and Marine Research, Bremerhaven, 27570 Germany; 3 Institute for Quantitative and Theoretical Biology, Heinrich Heine University Düsseldorf, Düsseldorf, 40225 Germany; 4 Deep-Sea Ecology and Technology, Alfred Wegener Institute Helmholtz Centre for Polar and Marine Research, Bremerhaven, 27570 Germany; 5 Polar Biological Oceanography, Alfred Wegener Institute Helmholtz Centre for Polar and Marine Research, Bremerhaven, 27570 Germany; 6 School of Environmental Sciences, University of East Anglia, Norwich Research Park, Norwich, NR4 7TJ United Kingdom; 7 School of Computing Sciences, University of East Anglia, Norwich Research Park, Norwich, NR4 7TJ United Kingdom; 8 MARUM – Center for Marine Environmental Sciences, University of Bremen, Bremen, 28359 Germany

**Keywords:** Microbial ecology, Metagenomics, Biogeochemistry

## Abstract

The Arctic Ocean is experiencing unprecedented changes because of climate warming, necessitating detailed analyses on the ecology and dynamics of biological communities to understand current and future ecosystem shifts. Here, we generated a four-year, high-resolution amplicon dataset along with one annual cycle of PacBio HiFi read metagenomes from the East Greenland Current (EGC), and combined this with datasets spanning different spatiotemporal scales (Tara Arctic and MOSAiC) to assess the impact of Atlantic water influx and sea-ice cover on bacterial communities in the Arctic Ocean. Densely ice-covered polar waters harboured a temporally stable, resident microbiome. Atlantic water influx and reduced sea-ice cover resulted in the dominance of seasonally fluctuating populations, resembling a process of “replacement” through advection, mixing and environmental sorting. We identified bacterial signature populations of distinct environmental regimes, including polar night and high-ice cover, and assessed their ecological roles. Dynamics of signature populations were consistent across the wider Arctic; e.g. those associated with dense ice cover and winter in the EGC were abundant in the central Arctic Ocean in winter. Population- and community-level analyses revealed metabolic distinctions between bacteria affiliated with Arctic and Atlantic conditions; the former with increased potential to use bacterial- and terrestrial-derived substrates or inorganic compounds. Our evidence on bacterial dynamics over spatiotemporal scales provides novel insights into Arctic ecology and indicates a progressing Biological Atlantification of the warming Arctic Ocean, with consequences for food webs and biogeochemical cycles.

## Introduction

The Arctic Ocean is experiencing unprecedented changes as a result of climate warming, progressing nearly four times faster than the global average [1]. Of particular significance is the rapid decline in sea-ice extent and thickness [2, 3], with future projections indicating frequent ice-free summers by 2050 [4]. In the Eurasian Arctic, accelerated rates of sea-ice decline are associated with increasing volume and heat content of inflowing Atlantic water (AW) [5]. The expanding influence of AW in the Arctic Ocean, termed Atlantification, not only impacts hydrographic and physicochemical conditions, but also provides avenues for habitat range expansion of temperate organisms [6, 7].

The impact of climate change on biological communities has become increasingly apparent across the Arctic Ocean in recent decades. Elevated primary production in shelf seas has been attributed to declining sea-ice extent and increasing phytoplankton biomass [8], particularly in the Eurasian Arctic where Atlantification is driving a poleward expansion of temperate phytoplankton [7, 9]. Concurrently, phytoplankton phenologies are also changing, with secondary autumnal blooms now occurring in seasonally ice-covered areas [10]. This will have major consequences for the organic matter pool of the Arctic Ocean. Sea-ice dynamics play an important role in the availability of nutrients and organic matter in surface waters and the transport of carbon to the deep-sea [11–13]. At sea-ice margins, strong melt events result in intense stratification, which traps organic material in surface waters and delays vertical export [11].

Considering their role as primary degraders of organic matter and mediators of biogeochemical cycles, assessing the consequences of such changes for bacterial communities is essential to understand and predict alterations to ecosystem functioning. Recent studies have documented distinctions in bacterial communities between Atlantic- and Arctic-derived waters [14], and between sea-ice and seawater [15]. In addition, sea ice-derived dissolved organic matter (DOM) has been shown to stimulate rapid responses by bacterial taxa and significantly alter communities in incubation experiments [16, 17]. However, in order to gain a deeper understanding of potential shifts in Arctic Ocean microbial ecology, communities need to be studied over high-resolution temporal scales and across natural environmental gradients such as the Arctic–Atlantic interface.

The Fram Strait, the main deep-water gateway between the Arctic and Atlantic Oceans, is a key location for conducting long-term ecological research over environmental gradients and under changing conditions [18]. Fram Strait harbours two major current systems; the East Greenland Current (EGC), transporting polar water (PW) southwards, and the West Spitsbergen Current (WSC), transporting AW northward. The EGC accounts for the export of ~50% of freshwater and ~90% of sea-ice from the central Arctic Ocean and carries Arctic hydrographic signatures [19]. Large-scale recirculation of AW into the EGC continuously occurs, although the magnitude varies across latitudes and over time [20, 21]. The mixing of AW and PW in the marginal ice zone (MIZ) creates different hydrographic regimes reflective of Arctic, mixed and Atlantic conditions, which can harbour unique bacterial compositions [14, 22]. It has been predicted that future Atlantification of the Arctic may result in a shift towards temperate, Atlantic-type communities [14]. However, further assessments of microbial population dynamics across spatiotemporal scales are needed to validate such hypotheses.

Here, we performed a high-resolution analysis of the temporal variation of bacterial taxonomy and function in the MIZ (2016–2018) and the core-EGC (2018–2020), covering the full spectrum of ice cover, daylight and hydrographic conditions. Our study is embedded in the “Frontiers in Arctic Marine Monitoring” (FRAM) ocean observing framework that employs mooring-attached sensors and autonomous Remote Access Samplers (RAS) to continuously monitor physicochemical parameters and biological communities in the Fram Strait. We analysed four-year 16S rRNA gene amplicon data supplemented with an annual cycle of PacBio HiFi read metagenomes, expanding a previous assessment of microbial dynamics over a single annual cycle in the EGC [23]. We hypothesise that high AW influx and low sea-ice cover result in communities dominated by chemoheterotrophic populations that taxonomically and functionally resemble those of temperate ecosystems. Our study provides essential insights into the impact of changing conditions on microbial ecology and biogeochemical cycles in the Arctic Ocean.

## Methods

### Seawater collection and processing

Autonomous sample collection and subsequent processing proceeded as previously described [23]. Briefly, RAS (McLane, East Falmouth, MA) were deployed over four consecutive annual cycles between 2016 and 2020, with deployments and recoveries occurring each summer (2019–2020 mooring recovered in 2021). From 2016 to 2018, RAS were deployed in the MIZ (78.83° N −2.79° E) and from 2018 to 2020 in the core-EGC (79° N −5.4° E), with average sampling depths of 80 and 70 m, respectively. The depths were chosen to prevent contact with moving ice overhead. In weekly to fortnightly intervals (Supplementary Table S1), ~1 L of seawater was pumped into sterile plastic bags and fixed with mercuric chloride (0.01% final concentration). After RAS recovery, water was filtered onto 0.22 µm Sterivex cartridges directly frozen at −20 °C until DNA extraction.

### Amplicon sequencing and analysis

DNA was extracted using the DNeasy PowerWater kit (Qiagen, Germany), followed by amplification of 16S rRNA gene fragments using primers 515F–926R [24]. These primers perform well at recovering marine mock communities, and were recently suggested as optimal for studying Arctic microbial communities [24, 25]. Sequencing was performed on a MiSeq platform (Illumina, San Diego, CA) using 2 × 300 bp paired-end libraries according to the “16S Metagenomic Sequencing Library Preparation protocol” (Illumina). Reads were subsequently processed into amplicon sequence variants (ASVs) using DADA2 and the SILVA v138 database [26–28]. Analysis and plotting were performed in RStudio [29], primarily using the vegan [30], limma [31], mixOmics [32], ggplot2 [33] and ComplexHeatmap [34] packages. Briefly, community composition was compared using Bray-Curtis dissimilarities and distance-based redundancy analysis (dbRDA) with the functions *decostand* and *dbrda* in vegan, and visualised using ggplot2. The influence of environmental variables on community dissimilarity was determined through a stepwise significance test on the dbRDA using the *ordiR2step* and *anova.cca* functions in vegan. ASVs were assigned to distribution groups based on the frequency of detection over time.

Co-occurrence networks were calculated for MIZ and core-EGC samples separately using the packages segmenTier [35] and igraph [36]. Oscillation signals were calculated for each ASV per year based on Fourier transformation of normalised abundances and compared using Pearson’s correlations. Only statistically significant positive correlations were retained (adjusted *p*-value < 0.05 after correction using the FDR method [37]). Using a network robustness analysis, a correlation coefficient of 0.7 was determined as a strong co-occurrence. Below this value, removal of a single node would cause network disruption. Networks were constructed using the co-occurrences that passed the above thresholds, and visualised in Cytoscape [38] with the Edge-weighted Spring-Embedded Layout. Values of centrality and node betweenness were calculated using igraph.

### PacBio metagenome sequencing

Nine samples from the 2016–2017 annual cycle in the MIZ were selected for metagenomic sequencing, using the same DNA as for amplicon sequencing. Sequencing libraries were prepared following the protocol “Procedure & Checklist – Preparing HiFi SMRTbell Libraries from Ultra-Low DNA Input” (PacBio, Menlo Park, CA) and inspected using a FEMTOpulse. Libraries were sequenced on 8M SMRT cells on a Sequel II platform for 30 h with sequencing chemistry 2.0 and binding kit 2.0. The sequencing was performed together with samples of another project, such that seven samples were multiplexed per SMRT cell. On average, this resulted in 268,000 reads per metagenome, with an N50 of 6.8 kbp.

### Taxonomic and functional annotation of HiFi reads

The 2.4 million generated HiFi reads were processed through a custom taxonomic classification and functional annotation pipeline. The classification pipeline followed similar steps to previously published tools, but with some modifications. A local database was constructed based on protein sequences from all species-representatives in the GTDB r202 database [39]. Prodigal v2.6.3 [40] was used to predict open reading frames (ORFs) on HiFi reads, which were subsequently aligned to the GTDB-based database using Diamond blastp v2.0.14 [41] with the following parameters: --id 50 --query-cover 60 --top 5 --fast. After inspection of the hits, a second filtering step was performed: percentage identity of >65% and an e-value of <1^˗10^. Using Taxonkit v0.10.1 [42], the last common ancestor (LCA) algorithm was performed, resulting in a single taxonomy for each ORF. A secondary LCA was subsequently performed for all ORFs from the same HiFi read, generating a single taxonomy for each read. Functional annotation of HiFi reads was performed using Prokka [43] followed by a series of specialised databases. This included using blastp v2.11.0 [44] or HMMscan (HMMER v3.2.1) [45] against dbCAN v10 [46], CAZy (release 09242021) [47], SulfAtlas v1.3 [48], the Transporter Classification [49], MEROPS [50] and KEGG [51] databases along with sets of Pfam HMM family profiles for SusD and TonB-dependent transporter genes. Functional gene counts were normalised by the average sequencing depth of 16 universal, single-copy ribosomal protein genes per sample [52] – providing “per genome” counts. Genes enriched under high- and low-ice cover conditions were identified using ALDEx2 [53].

### Metagenome-assembled genome recovery

In order to maximise the recovery of metagenome-assembled genomes (MAGs), metagenomes were clustered into two groups based on dissimilarity in ASV composition of the corresponding amplicon samples. Samples were individually assembled using metaFlye v2.8.3 (parameters: --meta --pacbio-hifi –keep-haplotypes --hifi-error 0.01). Contigs with a length of <10 kbp were removed and the remaining contigs were renamed to reflect the sample of origin. Contigs from each group were concatenated into a single file. Coverage information, necessary for binning, was acquired through read recruitment of raw reads from all metagenomes to the contigs using Minimap2 v2.1 [54], using the ‘map-hifi’ preset. Contigs were binned using Vamb v3.0.2 [55] in multisplit mode using three different sets of parameters (set1: -l 32 -n 512 512, set2: -l 24 -n 384 384, set3: -l 40 -n 768 768). Completeness and contamination estimates of bins were determined using CheckM v1.1.3 [56], and those with >50% completeness were manually refined using the interactive interface of Anvi’o v7 [57]. A consensus set of refined MAGs with non-redundant contigs was obtained using DASTool v1.1.1 [58]. The consensus MAGs were de-replicated at 99% average nucleotide identity using dRep v3.2.2 [59] (parameters: -comp 50 -con 5 -nc 0.50 -pa 0.85 -sa 0.98), resulting in 47 population-representative MAGs. A phylogenetic tree was reconstructed that also incorporated MAGs recently published from the Fram Strait [22], following a procedure outlined previously [52]. Briefly, 16 single-copy universal ribosomal protein genes were identified in each MAG using HMMsearch against the individual Pfam HMM family profiles and aligned using Muscle v3.8.15 [60]. Alignments were trimmed using TrimAI v1.4.1 [61], concatenated, and submitted to FastTree v2.1.0 [62]. The tree was visualised and annotated in iToL [63].

### Classification, abundance and distribution of MAGs

A dual taxonomic classification of MAGs was performed using single-copy marker and 16S rRNA genes. Firstly, MAGs were assigned a taxonomy using the GTDBtk tool v1.7.0 [64] with the GTDB r202 database. Secondly, extracted 16S rRNA gene sequences were imported into ARB [65], aligned with SINA [66] and placed into the SILVA SSU 138 Ref NR99 reference tree using ARB parsimony. Those containing a 16S rRNA gene were linked to ASV sequences through competitive read recruitment using BBMap of the BBtools programme v35.14, with an identity threshold of 100%.

The distribution of MAGs across the Arctic Ocean were determined through recruitment of reads from the herein generated metagenomes and published datasets from the Tara Arctic and MOSAiC expeditions (Supplementary Table S11). Counts of competitively mapped reads were converted into the 80% truncated average sequencing depth, TAD80 [67]. Relative abundance was then determined as the quotient between the TAD80 and the average sequencing depth of 16 single-copy ribosomal protein genes. Ribosomal proteins were identified following the same procedure outlined above, and their sequencing depth estimated using read recruitment with minimap2 (for PacBio-derived metagenomes) and BBMap (for Illumina-derived metagenomes).

### Mooring and satellite data

Bacterial community data was placed into context using in situ measured environmental parameters (Supplementary Table S1). Temperature, depth, salinity and oxygen concentrations were measured using Seabird SBE37-ODO CTD sensors and chlorophyll *a* concentration was measured using a WET Labs ECO Triplet sensor, all attached to the RAS. Sensor measurements were averaged over 4 h around each sampling event. The relative proportions of AW and PW were determined as described previously [23]. Physical sensors were manufacturer-calibrated and processed in accordance with https://epic.awi.de/id/eprint/43137. Mooring-derived data are published under PANGAEA accession 904565 [68], 941159 [69], and 946539 [70]. Sea-ice concentrations, derived from the AMSR-2 satellite, were downloaded from https://seaice.uni-bremen.de/sea-ice-concentration-amsr-eamsr2, and averaged over a 15 km radius around the moorings.

## Results

**Fig. 1:**
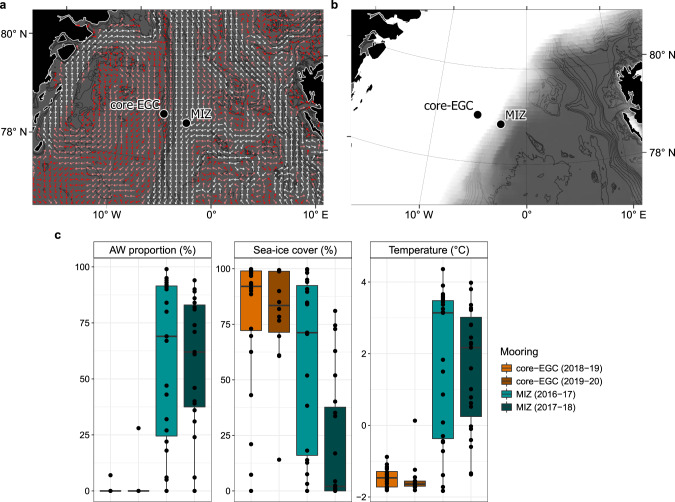
Location of seafloor moorings and environmental conditions in the MIZ (2016–2018) and core-EGC (2018–2020). **a** Example representation of monthly average (January 2020) current velocities at the approximate depth of sampling (78 m). White and dark red arrows indicate strongest and weakest velocities, respectively. **b** Example representation (December 2019) of sea-ice cover. Increasing opacity of white colour reflects increasing sea-ice cover (pure white = 100%). Current and sea-ice data were obtained from copernicus.eu under ‘ARCTIC_ANALYSIS_FORECAST_PHY_002_001_a’. **c** Variation in AW proportion, ice cover and water temperature at the two moorings. The bathymetric map was made using data from GEBCO.

The amplicon dataset incorporates samples (>0.2 µm fraction) collected at weekly to fortnightly intervals in the MIZ (2016–2018) and central EGC (core-EGC; 2018−2020) between 70 and 90 m depth (Supplementary Table S1). The two locations were selected in order to capture the full spectrum of water mass and sea-ice conditions. The core-EGC was characterised by year-round dense ice cover (hereon abbreviated as “high ice”) and PW conditions. In contrast, the MIZ featured variable, generally lower ice cover (hereon abbreviated as “low ice”) and periodic AW influx (Fig. 1). To visually portray this variability, animated GIFs were created for current velocities (Supplementary Fig. S1) and sea-ice cover (Supplementary Fig. S2) over the four-year period. Combining the high-resolution data from both mooring locations allowed for the assessment of bacterial community dynamics over time and in relation to Arctic- and Atlantic-dominated conditions.

### Bacterial community and population dynamics over time

**Fig. 2:**
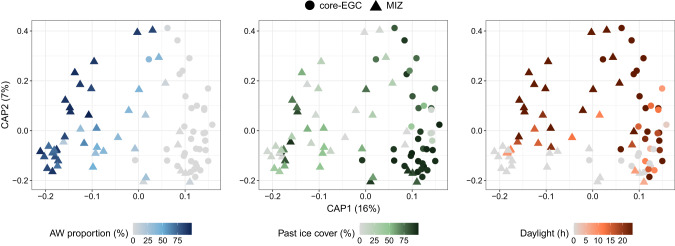
Community structure across water mass, sea-ice and daylight conditions. Distance-based redundancy analysis based on Bray-Curtis dissimilarities of community composition along with AW proportion (blue), past ice cover (green) and daylight (orange) as constraining factors. The factors were selected using a stepwise significance test and combined into a single model (*R*^*2*^ = 0.1, *p* = 0.01) that constrains 14% of the total variation. For ease of interpretation, the environmental conditions are visualised individually on the same ordination.

The amplicon dataset encompasses 12.5 million quality-filtered reads in 84 samples, with an average of 134,588 reads per sample. A total of 4083 ASVs (Supplementary Table S2) were recovered, which were initially used in a taxonomy-independent approach to assess community dynamics over environmental gradients (Fig. 2). A dbRDA with stepwise significance testing identified AW proportion, daylight and past ice cover (average ice cover of the days preceding the sampling event) as the significant factors constraining compositional variation (model *R*^2^ = 0.23, *p* = 0.001). AW proportion explained 13% of the variation in bacterial community dissimilarity, compared to 6% for daylight and 4% for past ice cover.

**Fig. 3:**
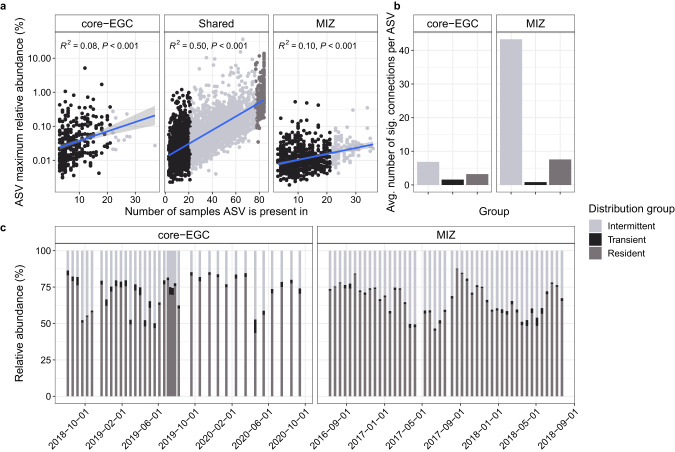
Distribution dynamics and co-occurrence of ASVs. **a** Occurrence of ASVs across samples in relation to their maximum relative abundances, along with categorisation into resident, intermittent and transient. **b** Average number of connections within the co-occurrence networks for resident, intermittent and transient ASVs. **c** Relative abundance dynamics of resident, intermittent and transient ASVs over time.

Assessing ASV dynamics at the two mooring locations over time revealed several distinct patterns. In total, 75% of the ASVs were detected at both mooring sites (i.e. shared), whilst 16% and 9% were unique to the MIZ and core-EGC respectively. The frequency of detection and maximum relative abundance of shared ASVs exhibited a strong positive linear relationship, i.e., those identified in more samples also reached higher maximum relative abundances (Fig. 3a). To better understand the structuring of communities and distinguish between ecologically different fractions, we categorised ASVs into three groups: (a) Resident (Res-ASVs), present in >90% of samples, (b) Intermittent (Int-ASVs), present in 25–90% of samples, and (c) Transient (Trans-ASVs), present in <25% of samples (Supplementary Table S3). Res-ASVs represented a small fraction of the diversity (231 ASVs) but the largest proportion of the sampled bacterial communities (43–87% relative abundance). In comparison, the 1943 Int-ASVs constituted 12–53% and the 1909 Trans-ASVs 0.4–9.3% of relative abundances. Presence of a dominant resident microbiome, represented by a minority of ASVs, is consistent with multiannual observations in the Western English Channel and Hawaiian Ocean time-series [71, 72].

Temporal dynamics of the three community fractions was linked to changes in AW proportion, evidenced by negative correlations for the resident (Pearson’s coefficient: −0.29, *p* < 0.05) and transient fractions (Pearson’s coefficient: −0.36, *p* < 0.01) compared to positive correlations for the intermittent fraction (Pearson’s coefficient: 0.37, *p* < 0.01). This is reflected in the more stable temporal dynamics at the core-EGC with less AW influence, compared to the MIZ (Fig. 3c). In addition, the transient fraction was positively correlated with ice cover (Pearson’s coefficient: 0.26, *p* < 0.05).

The dynamics of the three community fractions were supported by co-occurrence networks computed at ASV level (Supplementary Fig. S3). The MIZ network contained more ASVs and more significant co-occurrences compared to the core-EGC, primarily driven by Int-ASVs. There were 283 more Int-ASVs in the MIZ than in the core-EGC network, and the number of connections per ASV was nine-fold higher. In contrast, Trans-ASVs were threefold more numerous and exhibited threefold more connections per ASV in the core-EGC compared to the MIZ network (Fig. 3b and Supplementary Information). Res-ASVs were comparable in number in both networks.

The resident microbiome was phylogenetically diverse, incorporating both abundant and rare community members. Res-ASVs were assigned to 61 families and 79 genera, with the *Flavobacteriaceae* (*n* = 15)*, Magnetospiraceae* (*n* = 13)*, Marinimicrobia* (*n* = 11), SAR11 Clade I (*n* = 21) and SAR11 Clade II (*n* = 17) harbouring the largest diversity. Maximum relative abundances of Res-ASVs ranged from 0.04 to 13.9%, with the most prominent being affiliated with SAR11 Clade Ia (asv1; 14%), *Polaribacter* (asv6; 14%), *Aurantivirga* (asv7; 12%), SUP05 (asv2; 12%), SAR92 (asv16; 11%) and SAR86 (asv3; 9%). Pronounced fluctuations of the intermittent community coincided with AW influx in the MIZ. Int-ASVs were more phylogenetically diverse than Res-ASVs, encompassing 254 genera, and included rare and abundant populations that reached 0.004–36% maximum relative abundance. The most diverse taxa included the SAR11 Clade II (*n* = 148), *Marinimicrobia* (*n* = 129), NS9 Marine Group (*n* = 78), AEGEAN-169 (*n* = 73), and SAR86 (*n* = 47). Those with largest relative abundances were affiliated with *Colwellia* (asv10; 36%), *Luteolibacter* (asv24; 15%), *Flavobacterium* (asv140; 10%), and *Polaribacter* (asv206; 10%). The resident and intermittent community fractions shared 71 genera, constituting 90% of the genus-level diversity of the resident microbiome. Hence, compositional changes over temporal scales relate to dynamics on the (sub-)species level.

### Taxonomic signatures of distinct environmental conditions

**Fig. 4:**
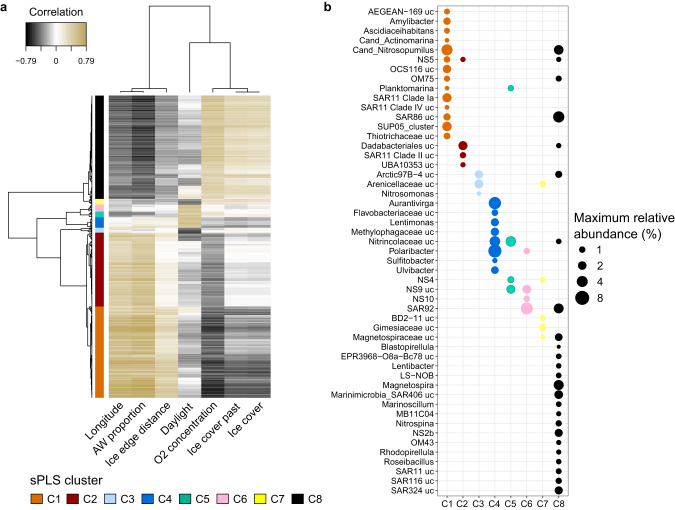
Sparse partial least square regression (sPLS) linking community structure and environmental parameters. **a** Heatmap showing eight major sPLS clusters, encompassing 430 ASVs with significant correlations to environmental conditions. **b** Representation of the most prominent genera per cluster. ASVs with <1% relative abundance were excluded, whilst the remaining were grouped by genus and the maximum abundance of each genus shown. Due to high collinearity with AW proportion, temperature and salinity were excluded. Thresholds: coefficients > 0.4, *p* < 0.05.

A sparse partial least squares regression analysis (sPLS) identified 430 ASVs that were associated with distinct environmental conditions. Based on similar, significant correlations (Pearson’s coefficient >  0.4, *p* < 0.05) to environmental parameters, the ASVs were grouped into eight distinct clusters (Fig. 4a and Supplementary Table S3), each comprising unique taxonomic signatures (Fig. 4b). The three largest clusters encompassed 88% of the ASVs, and were distinguishable based on their associations to different water mass and ice cover conditions. Clusters C1 and C2 represent AW conditions, with C1 also being associated with low-ice cover. In contrast, cluster C8 represents PW conditions under high-ice cover. In accordance with the distribution dynamics described above, the AW-associated clusters comprised a higher proportion of Int-ASVs, 51–88%, compared to ~50% Res-ASVs in PW-associated clusters. Five smaller clusters (C3–C7) correspond to polar day and night under different ice cover and water mass conditions. Comparing the most prominent ASVs (>1% relative abundance) of each cluster revealed unique taxonomic signatures at the genus level (Fig. 4b). For instance, *Amylibacter*, SUP05 and AEGEAN-169 are signatures of the AW-associated, low-ice cluster C1, whereas SAR324, NS2b and *Magnetospira* are signatures of the PW-associated, high-ice cluster C8. Overall, this pattern underlines that water mass and ice cover have the largest influence on microbial community structure, with a smaller number of ASVs being influenced by daylight and seasonality.

### MAGs and comparison to other Arctic datasets

Nine PacBio HiFi read metagenomes spanning one annual cycle in the MIZ yielded 43 manually refined, population-representative MAGs, delineated at 99% ANI (Supplementary Table S4). The MAGs were of medium- and high-quality according to MIMAG standards [73], exhibited low fragmentation (average number of contigs = 33), and >80% contained at least one complete rRNA gene operon. MAGs covered a broad phylogenetic diversity, including 35 genera, 27 families and nine classes (Supplementary Fig. S4). For deeper ecological insights, we contextualised ASV dynamics with MAGs to link distribution with metabolic potential. Of the 27 ASVs linked to a MAG through competitive read recruitment (100% identity threshold), 18 were associated with sPLS clusters and thus distinct environmental conditions – these are hereon referred to as “signature populations” (Supplementary Table S5). Signature populations included some of the most abundant ASVs, such as asv6-*Polaribacter* and asv7-*Aurantivirga* from cluster C4 (polar day-associated) and asv18-SAR86 from cluster C8 (high-ice, PW-associated).

**Fig. 5:**
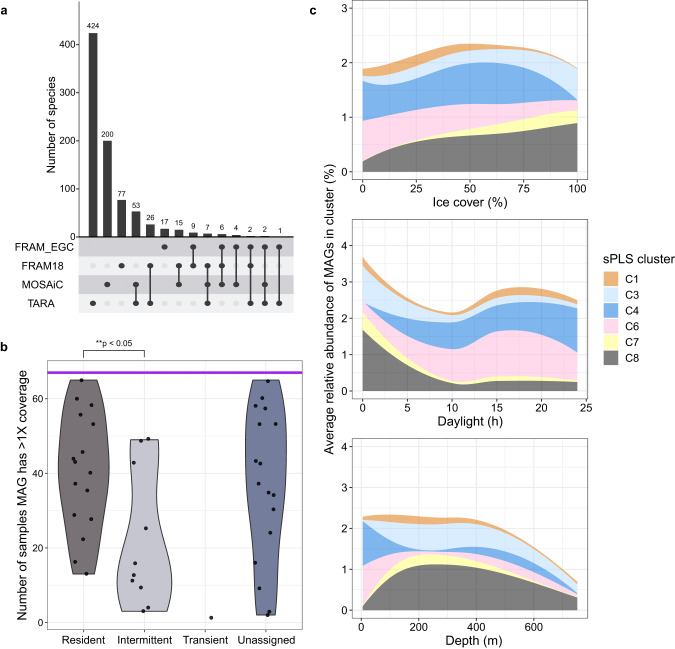
Comparison and dynamics of MAGs across the Fram Strait and Arctic Ocean. We compared metagenome-assembled genomes (MAGs) generated in this study (FRAM_EGC), and from samples previously collected in the Fram Strait (FRAM18) [23], in the Arctic Ocean during summer (TARA) [34], and in the Arctic Ocean during winter (MOSAIC) [35]. **a** Number of shared and unique species across the four MAG datasets, determined by comparisons at 95% average nucleotide identity threshold. **b** Number of metagenomes in which FRAM_EGC MAGs were detected with at least 1× coverage. The horizontal purple line represents the total number of samples (*n* = 67). **c** Average relative abundance of sPLS clusters (Fig. 4) across different ice cover, daylight and depth values determined by read recruitment from Arctic Ocean and Fram Strait metagenomes to the respective FRAM_EGC MAGs. The ~4000 m sample from MOSAiC was not included.

To corroborate the associations of signature populations with distinct environmental conditions, we assessed their spatiotemporal dynamics across the Arctic Ocean. This comparison included an additional 59 metagenomes as well as 1184 MAGs and metagenomic bins from the Fram Strait [22], the Tara Arctic expedition (TARA) [74], and the MOSAiC expedition [75]. Combined, these datasets provide an extensive geographical and seasonal coverage, from above the continental shelf in summer to the central basin in winter. Combining the MAG datasets resulted in 843 species-level clusters at 95% ANI (Supplementary Table S6). Each dataset comprised a mixture of unique and shared species (Fig. 5a), but there were no cosmopolitan species. Of the MAGs recovered in this study, hereon termed FRAM_EGC MAGs, 42% were unique species. However, these results are influenced by differences in dataset size, sequencing platforms and analysis pipelines, e.g. co-assembly (TARA) vs. single sample assembly (FRAM and MOSAiC).

FRAM_EGC MAGs were among the most abundant and widely detected (Supplementary Table S7) across the Fram Strait and Arctic Ocean, constituting 0.02–58% of bacterial communities. Their distribution across the wider Arctic supported the dynamics observed in the EGC; e.g. residents (associated with a Res-ASV) were more widely detected than intermittent or transient populations (Fig. 5b, Supplementary Figs. S5 and S6). Three of the resident FRAM_EGC MAGs, one assigned to OM182 (UBA9659) and two to *Thioglobus*, were detected in >90% of all metagenomes. One of these species did not have a MAG representative in the other Arctic datasets, highlighting that our study contributes novel genomic information towards a better understanding of Arctic Ocean microbial ecology. Furthermore, the dynamics of signature-population MAGs across the Arctic supported their association with distinct environmental conditions. MAGs from cluster C8 (high-ice and PW) and C7 (high-ice and polar night) reached higher relative abundances in mesopelagic depths (TARA) and during polar night (MOSAiC) (Fig. 5c). In contrast, a higher relative abundance of C4 and C6 (polar day) MAGs occurred in surface water collected during summer (TARA).

### Functional potential of Atlantic and Arctic signature populations

Connecting ASV temporal dynamics and MAG functional potential facilitated predictions on the ecology of signature populations within the context of environmental conditions. Of particular interest were the signature populations of Atlantic (cluster C1) and Arctic (cluster C8) conditions, as they could provide insights into how bacterial community structure and function may shift in the future Arctic Ocean. Comparing the functional potential of MAGs revealed that Atlantic and Arctic signature populations clearly differ in substrate metabolism. In short, signature populations of Arctic conditions harboured genes for autotrophy and the utilisation of bacterial- and/or terrestrial-derived compounds, compared to Atlantic signature populations that were functionally connected to phytoplankton-derived organic substrates. Ecological descriptions of all signature populations and functional gene tables are provided in Supplementary Information and Supplementary Files S1, respectively.

#### Atlantic signature populations

**Fig. 6:**
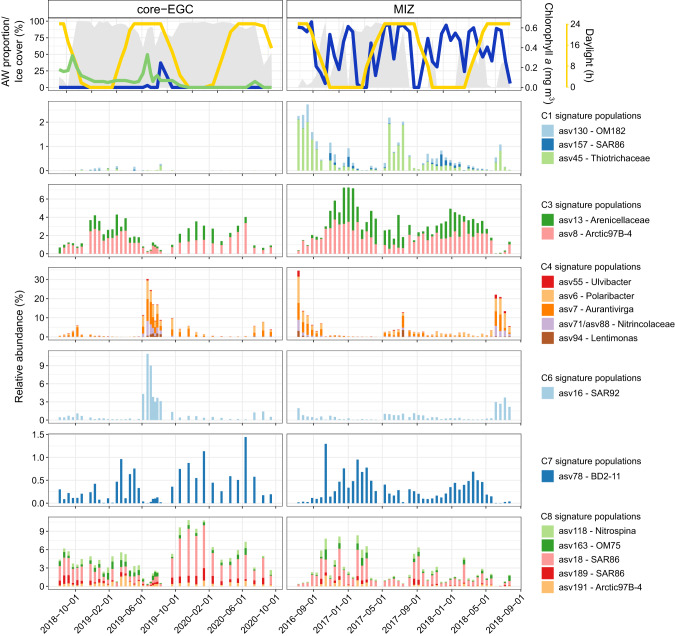
Temporal dynamics of signature populations. Signature populations were identified as ASV representatives from sPLS clusters that a corresponding MAG was recovered for (based on 100% identity threshold competitive read recruitment). The temporal dynamics visualised are derived from ASV data. The missing chlorophyll data in 2016−2018 is due to the lack of a sensor on the MIZ mooring.

Atlantic signature populations included *Thiotrichaceae* (asv45), OM182 (asv130) and SAR86 (asv157) from the *Gammaproteobacteria* (Fig. 6). Although all three populations were more abundant in the MIZ, differences were observed in their temporal dynamics (asv45 peaking during polar day, asv157 peaking during polar night, and asv130 showing minimal seasonality). The asv45 and asv130 populations both harboured genes for the degradation of phytoplankton-derived organic compounds. For asv45-*Thiotrichaceae*, this included the capacity to oxidise methanethiol (MTO gene) and the downstream reaction products, sulfide (*dsrAB* and *soeABC*) and formaldehyde (H4-MPT-dependent oxidation pathway), which could provide carbon, sulfur and energy. The asv130-OM182 population encoded a more diverse substrate metabolism, with the capacity to use dissolved organic sulfur (DOS) and nitrogen (DON) compounds, such as taurine and methylamine, as well as carbon monoxide (CO) as supplemental energy source. The capacity to store and use elemental sulfur was evidenced by a polysulfide reductase and flavocytochrome c-sulfide dehydrogenase. Together with its flagellar machinery, this suggests a motile, heterotrophic, carboxydovorous lifestyle.

#### Arctic signature populations

Arctic signature populations included *Nitrospina* (asv118), OM75 (asv163), SAR86 (asv18 and asv189) and Arctic97B-4 (asv191) affiliated with cluster C8, as well as BD2-11 (asv78) affiliated with cluster C7 (high-ice and polar night) (Fig. 6). Their metabolic potential and predicted ecological role varied considerably. The most prominent population (asv18-SAR86), reaching 8% relative abundance, harboured a heterotrophic metabolism with the capacity to gain supplemental energy through a green-light proteorhodopsin. Although similar to other SAR86 members [76, 77], asv18-SAR86 has an enriched repertoire of peptidases (*n* = 19) compared to carbohydrate-active enzymes (*n* = 7), as well as genes for D-amino acid metabolism.

Two of the Arctic signature populations were affiliated with enigmatic taxa, including the Arctic97B-4 (*Verrucomicrobiae; Pedospharaceae*) and BD2-11 (*Gemmatimonadota*). Arctic97B-4 was shown to be enriched in the particle-attached fraction in the Southern Ocean [78] and in subsurface waters [79, 80]. In comparison, BD2-11 has largely been observed in terrestrial and freshwater environments or in deep-sea sediments [81]. The genomic content of the Arctic97B-4 population indicated a motile chemomixotrophic lifestyle with the capacity to fix carbon, assimilate sulfate, and synthesise the vitamins riboflavin and biotin. This population encoded a high number of CAZymes (23 genes) and sulfatases (84 genes). The most numerous CAZyme gene families are involved in animal glycan degradation, such as sialic acids (GH33). The BD2-11 population encodes genes for inorganic and organic compound metabolism, including aerobic denitrification (*nap, nirK*) and the metabolism of taurine, hypotaurine, D-amino acids, dicarboxylic acids and halogenated haloaliphatic compounds.

### Whole-community functional shifts with contrasting environmental conditions

The raw HiFi reads contained 17.6 million ORFs (Supplementary Table S8), with 54% being assigned a function and 92% a taxonomy. Expectedly, taxonomic classifications varied in resolution, with 92% of genes assigned to a kingdom and 37% to a genus (Supplementary Fig. S7). Evident taxonomic shifts over the annual cycle included higher proportions of *Bacteroidia* during polar day and low-ice cover; compared to *Verrucomicrobiae*, BD2-11 and *Marinimicrobia* under polar night and high-ice cover, in agreement with ASV dynamics. A dissimilarity analysis of community functionality separated samples into two distinct clusters, with ice cover being the only statistically significant factor between the two (F-statistic = 12.6, *p* = 0.009) (Supplementary Fig. S8). A total of 1088 differentially abundant genes were identified between the two clusters, with 328 and 845 genes enriched under high- and low-ice conditions, respectively.

#### Enriched functions under different ice-cover regimes

**Fig. 7:**
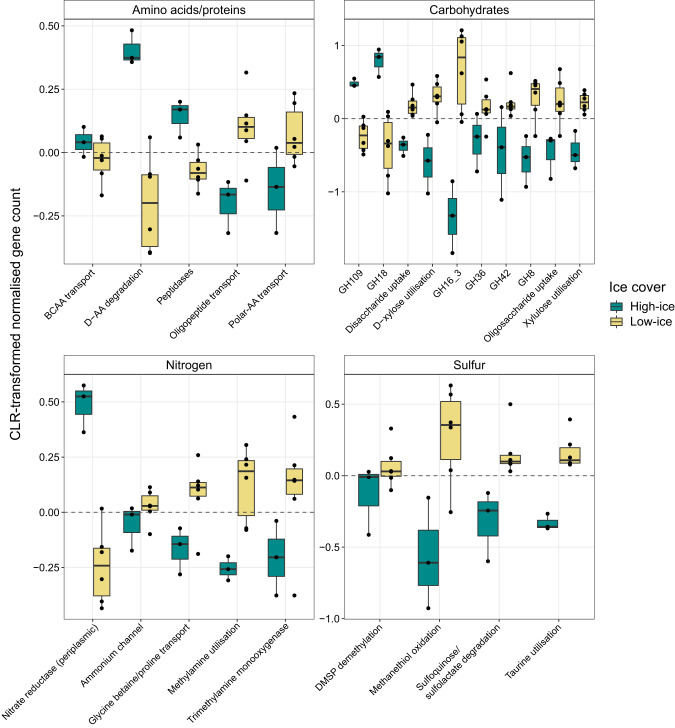
Selected genes involved in the metabolism of organic and inorganic compounds enriched under high- and low-ice conditions. Enrichment is displayed as centred-log ratio transformed normalised gene counts. Where several genes of a single pathway or mechanism were identified as enriched, they were grouped into one and the term ‘utilisation’ used (e.g. “taurine utilisation” indicates the uptake and degradation of taurine). When single genes were identified, the corresponding gene names are included. AA amino acids, BCAA branched-chain amino acids, GH glycoside hydrolase.

In agreement with Arctic and Atlantic signature populations, the enrichment of genes under high- and low-ice cover suggested differences in substrate utilisation (Supplementary Fig. S9). Low-ice communities were enriched in genes involved in the utilisation of phytoplankton-derived carbohydrates as well as DON and DOS compounds, including dimethylsulfoniopropionate (DMSP), taurine, sulfoquinovose and methylamine (Fig. 7). In addition, glycoside hydrolase families involved in the degradation of laminarin, α-galactose- and β-galactose-containing polysaccharides (GH16, GH36, GH42 and GH8), and genes related to the metabolism of mono- and disaccharides, such as D-xylose, glucose and rhamnose, were enriched (Fig. 7). All of these compounds have been related to phytoplankton production [82] and can act as carbon, nitrogen and sulfur sources for heterotrophic microbes [83, 84].

Under high-ice cover, 50% fewer genes were enriched, and they were mostly related to the recycling of bacterial cell wall carbohydrates, proteins, amino acids, aromatics and ketone compounds (Fig. 7). Reduced phytoplankton productivity under high-ice cover and during polar night [23] limits the availability of fresh labile organic matter, which would necessitate alternative growth strategies. For instance, the enrichment of an assimilatory nitrate reductase gene (*nap*) indicates a need for utilising inorganic nitrogen compounds. Enrichment of GH109 and GH18 involved in peptidoglycan and chitin degradation [85], along with genes for D-amino acid degradation, indicate an increased reliance on recycling of bacterial-derived organic matter. Furthermore, we observed an enrichment in genes for the degradation of aromatic and ketone compounds, such as phenylpropionate (Fig. 7).

## Discussion

**Fig. 8:**
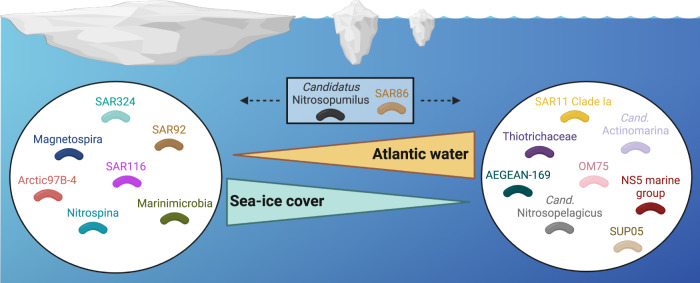
Bacterial communities under contrasting AW influx and ice cover conditions. Illustration showing the ten taxonomic groups with highest average relative abundances under Atlantic vs. Arctic conditions, derived from the relative abundances of Int-ASVs (sPLS cluster C1) and Res-ASVs (sPLS cluster C8), respectively. Figure was generated using Biorender.com.

In recent decades, the Atlantic influence in the Arctic Ocean has expanded, a process termed Atlantification [5, 6]. Atlantification encompasses the multi-faceted physicochemical impacts of northward-flowing AW, such as accelerated sea-ice decline, weakened water column stratification and altered nutrient availability. Although its impact on microbial communities has been postulated [14], we provide the first high-resolution analysis over a natural mixing zone between outflowing PW and inflowing AW in Fram Strait to assess potential ecological implications. We show that sea-ice cover and AW influx have a considerable impact on the composition, structure and functionality of bacterial communities. Densely ice-covered PW harboured a temporally stable, resident microbiome capable of using versatile substrates, with an enriched potential to degrade bacterial- and terrestrial-derived substrates as well as inorganic compounds. In contrast, low ice cover and high AW influx coincided with seasonally fluctuating populations that are functionally linked to phytoplankton-derived organic matter. We further identified bacterial signatures of distinct environmental conditions in the EGC (Fig. 8), showed the consistency of these patterns across the wider Arctic Ocean, and assessed ecological roles through MAG-based functional gene content. Our combined population- and community-level evidence suggests a future “Biological Atlantification” of the Arctic Ocean.

### Bacterial communities under different water mass and ice cover regimes

The pronounced impact of AW influx reflects the role of water masses as physical barriers to and conduits of dispersion for planktonic organisms. Influx events thus result not only in physiochemical changes, but also the mixing of microbial communities. How the microbiomes are reshaped under these events is a function of the degree of influx as well as the size (in number), competitive fitness and physiological adaptations of individual populations. Our dataset reveals that large influx events over short timescales can lead to the “replacement” of populations, evidenced by the dominance of AW-derived populations (Int-ASVs) in the MIZ. In contrast, the core-EGC, with rare occurrences of AW influx, harboured a temporally stable resident community that is adapted to polar conditions and constantly seeded from southward-flowing PW. However, the continual detection of the resident community in the MIZ indicates that even large influx events do not result in complete community turnover. Although the hydrological dynamics assessed here are more rapid than the gradually proceeding Atlantification of other Arctic regions, northward advection of organisms and subsequent replacement has already been documented for phyto- and zooplankton [7, 9, 86].

In addition to AW influx, bacterial communities were significantly impacted by sea-ice cover, which reflects its integral role in shaping Arctic Ocean ecosystems. Of particular significance is the influence of sea ice on water column stratification and organic matter availability. Sea ice supports rich biological communities that contribute significantly to Arctic Ocean primary production and the pool of organic matter [87, 88]. The melting of sea ice results in the release of dissolved and particulate organic matter, which heterotrophic bacteria can be highly responsive to [16, 17, 89]. However, ice-derived meltwater also induces rapid and strong stratification of the water column, which can reduce the mixed layer depth to as little as 5 m [11]. This shallow mixed layer can support prolonged phytoplankton blooms, but also trap the produced organic carbon, delaying vertical export [11]. In contrast, ice-free conditions result in a deeper mixed layer, shorter but more pronounced phytoplankton blooms and a higher response of grazers [11], potentially contributing to an increased availability of organic carbon to communities below. Considering the sampling depth in this study (70–80 m), the bacterial communities likely experienced an indirect influence from sea ice, through its impact on mixed layer depth, mixing and the vertical export of surface water production.

AW influx and sea-ice cover are intrinsically linked in the Eurasian Arctic. Consequently, the majority of signature populations were associated with either Arctic (high-ice and low-AW) or Atlantic (low-ice and high-AW) conditions. Furthermore, these populations were metabolically distinguishable, with Arctic signature populations harbouring genes for chemoautotrophy and the utilisation of bacterial and/or terrestrial-derived compounds. In the Beaufort Sea and Canadian Arctic, heterotrophic *Alphaproteobacteria* (*Rhodobacterales* and *Rhodospirillales*) and SAR324 (Chloroflexi) were shown to encode [90] and transcribe [91] pathways for the degradation of terrestrial-derived aromatic compounds. Similarly, Royo-Llonch et al. [74] described a number of bacteria as Arctic habitat specialists with versatile metabolisms, including the potential for autotrophy and denitrification. In this study, we found further examples of specific adaptations to Arctic Ocean conditions. For instance, the asv18-SAR86 population appears adapted towards proteinaceous and bacterial-derived compounds, with a reduced capacity for carbohydrate degradation compared to other SAR86 [76, 77]. In addition, the asv78-BD2-11 population encodes the capacity to use diverse inorganic and organic substrates, indicating a high degree of metabolic flexibility. The metabolic distinctions of Arctic signature populations illustrate evolutionary adaptations to the unique hydrological and physicochemical conditions. The Arctic Ocean is characterised by a comparatively large terrestrial and riverine influence [92, 93] and experiences a short productive season with a single phytoplankton bloom, compared to biannual bloom events in temperate oceans. This results in an organic matter pool rich in terrestrial-derived material, up to 33% in the case of DOM [94], which has likely contributed to the enrichment of distinct metabolic potentials.

Atlantic signature populations featured a closer relation to labile, phytoplankton-derived organic matter. For example, the asv45-*Thiotrichaceae* population harbours genes for the degradation of methanethiol and its downstream reaction products. Methanethiol originates from DMSP demethylation [95], an osmoprotectant produced by phytoplankton. DMSP concentrations in the Arctic Ocean are spatially heterogeneous and influenced by water mass and sea ice, with highest concentrations in areas with AW inflow [96] where its availability is tightly coupled to chlorophyll [96, 97]. Methanethiol concentrations would thus be elevated in AW during polar day. Similarly, the concentration of CO and its production by phytoplankton is also elevated in temperate compared to Arctic water masses [98]. The asv130-OM182 population encodes genes for CO degradation, as well as a capacity to use DOS compounds and store sulfur that may contribute to sustaining its more stable temporal dynamics in the MIZ. Given that previous reports of such a metabolism are restricted to members of the *Roseobacter* clade [99], the asv130-OM182 population may contribute to connecting carbon and sulfur cycles.

### Connectivity and structuring of bacterial communities across the Arctic

The identification of signature populations not only highlights ecological distinctions associated with different environmental regimes, but also aids in elucidating patterns in dispersal and connectivity in the Arctic Ocean. Although microbial species have been previously associated with certain depth layers and regions in the Arctic [74], this is the first study to evidence tight associations of populations with specific conditions over seasonally and geographically resolved scales. For example, polar day signatures found in the MIZ between 2016 and 2018 were also abundant in surface waters above continental shelves during the summer of 2013 (TARA). In addition, the here identified polar night and high-ice signature populations, which are of particular significance due to limited sampling of these conditions, were also abundant in the central Arctic during winter (MOSAiC). The consistency in dynamics of signature populations over space and time indicates a strong connectivity between Arctic regions, which is in agreement with the relatively short residence times of upper water layers [100]. Consequently, local environmental forcing is likely the key process shaping microbial communities. Furthermore, the prevalence of Arctic winter signatures in mesopelagic depths during summer suggests that solar- and meltwater-induced stratification contribute to shaping bacterial distribution.

In the core-EGC, where conditions are temporally stable, we identified a persistent, resident community fraction. The temporal stability of resident populations in the core-EGC, their variable dynamics in the MIZ, and low detection rate across summer Arctic samples suggests an adaptation to high-ice and PW conditions. However, in order to persist, the populations must be continually seeded from southward-flowing PW, underlining high dispersal and connectivity in the Arctic. Although the presence of a persistent community fraction has been reported from the Western English Channel and Hawaiian Ocean time-series [71, 72], this is the first such description from the Arctic. It is also a feature likely restricted to the central Arctic and core-EGC, as bacterial communities of continental shelf and peripheral regions are exposed to more dynamic conditions and stronger seasonal forcing.

### Biological Atlantification and future Arctic Ocean bacterial communities

Considering population- and community-level dynamics in concert with contrasting environmental conditions, we predict a Biological Atlantification of Arctic Ocean bacterial communities. Biological Atlantification will be driven by northward advection of populations, coupled with shifting physicochemical conditions from expanding AW influence as well as its associated effects on primary producers and higher trophic levels. There are two underlying mechanisms; “replacement” through advection, mixing and species sorting (as outlined above), and physiological or evolutionary adaptation. We hypothesise that replacement will be more commonplace for bacteria with narrow ecological niches due to their sensitivity to change. In addition, replacement is more likely to occur in the central Arctic and above Eurasian shelves. With ice-free summers predicted by 2050, the central Arctic is shifting to a seasonally dynamic environment. This will reduce the niche space of bacteria that are adapted to permanent ice cover, while benefitting those adapted to conditions of the shelf and peripheral regions. Similarly, the Eurasian shelves will experience the immediate impact of Atlantification along with the northward expansion of temperate species. In short, we envision a net shift in bacterial distribution from shelf regions to the central Arctic, and from the North Atlantic onto the Eurasian Arctic shelves. However, adaptation will also play a role in the reshaping of communities, but will likely be more commonplace among bacteria with wider ecological niches and higher competitive fitness that are less vulnerable to changing conditions (Fig. 8).

## Supplementary information

Supplementary Figure S1

Supplementary Figure S2

Supplementary Figure S3

Supplementary Figure S4

Supplementary Figure S5

Supplementary Figure S6

Supplementary Figure S7

Supplementary Figure S8

Supplementary Figure S9

Supplementary Information

Supplementary Tables

Supplementary Files

## Data Availability

The 16S rRNA gene sequences are available at EBI-ENA under PRJEB43890 (2016−17), PRJEB43889 (2017−18), PRJEB54562 (2018−19), and PRJEB54586 (2019−20). Individual sample accessions are provided in Supplementary Table S9. The metagenomic sequence data and MAGs generated are available at EBI-ENA under PRJEB52171 (accessions provided in Supplementary Table S10). Tara Arctic data are available under PRJEB9740. MOSAiC accession numbers are shown in Supplementary Table S11. Functional gene annotations for all signature populations are provided in Supplementary Files S1. Physicochemical parameters are available under PANGAEA accessions 904565 [68], 941159 [69], and 946539 [70].

## Code availability

Bioinformatic code for reproducing analyses and generating figures, along with necessary data files, is available at https://github.com/tpriest0/FRAM_EGC_2016_2020_data_analysis.
