# Supplementary figures and images for: Atlantic water influx and sea-ice cover drive taxonomic and functional shifts in Arctic marine bacterial communities
Source: ISME J. 2023 Jul 8;17(10):1612–25. doi: 10.1038/s41396-023-01461-6 (PMC10504371; doi:10.1038/s41396-023-01461-6)

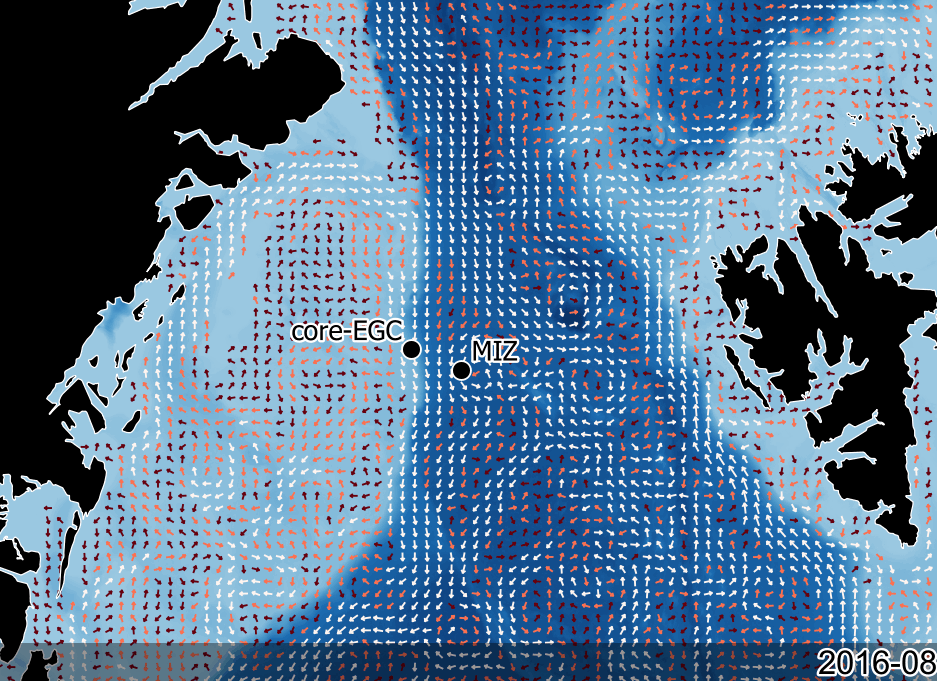

Supplement: Supplementary file 1 — Supplementary Figure S1 [file 41396_2023_1461_MOESM1_ESM.gif]

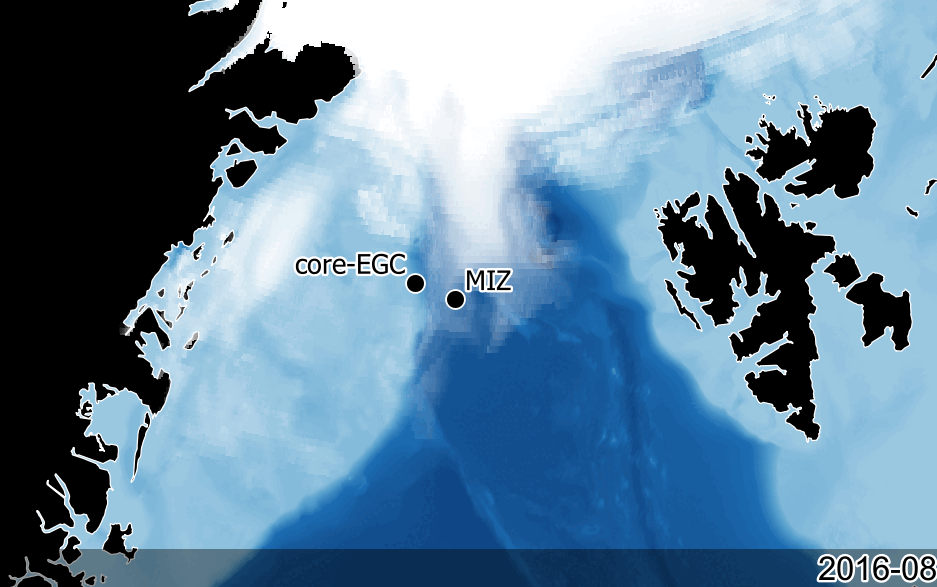

Supplement: Supplementary file 2 — Supplementary Figure S2 [file 41396_2023_1461_MOESM2_ESM.gif]

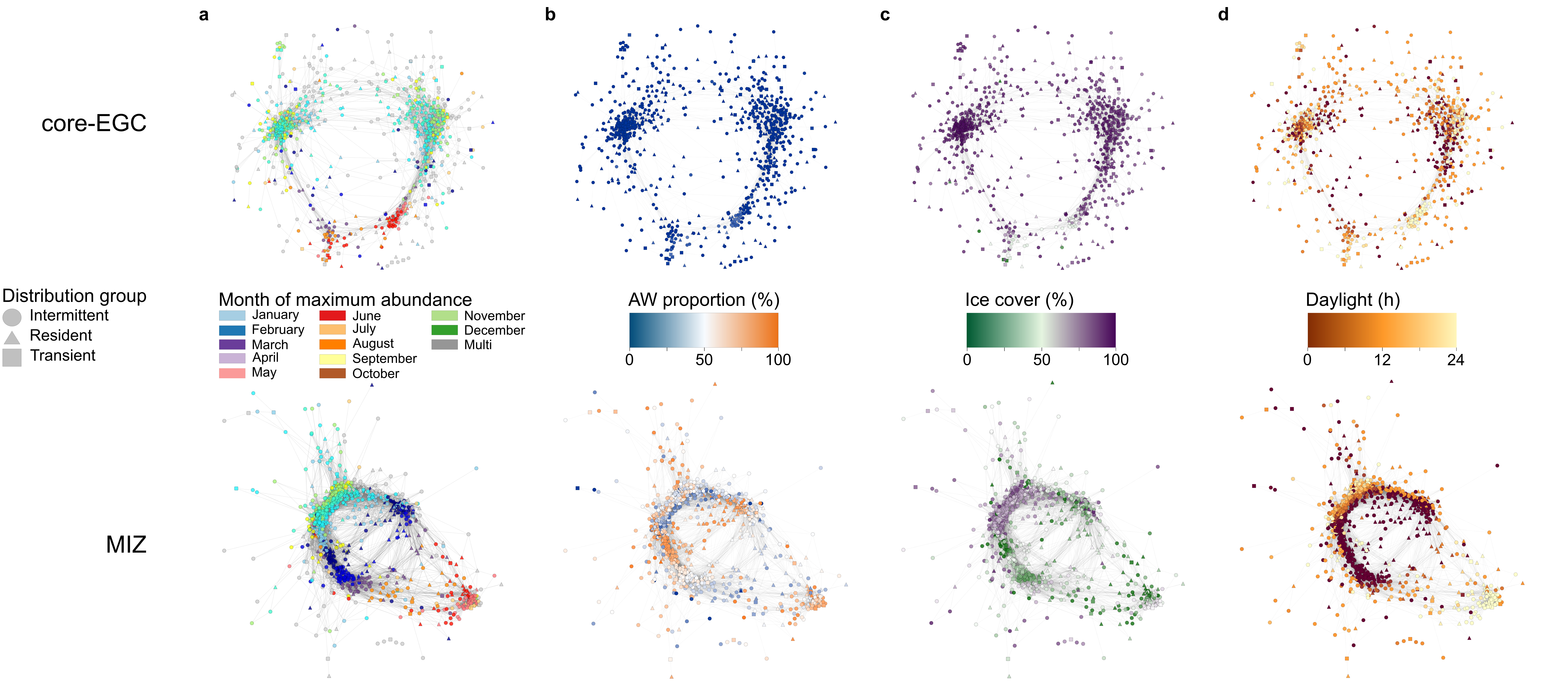

Supplement: Supplementary file 3 — Supplementary Figure S3 [file 41396_2023_1461_MOESM3_ESM.png]

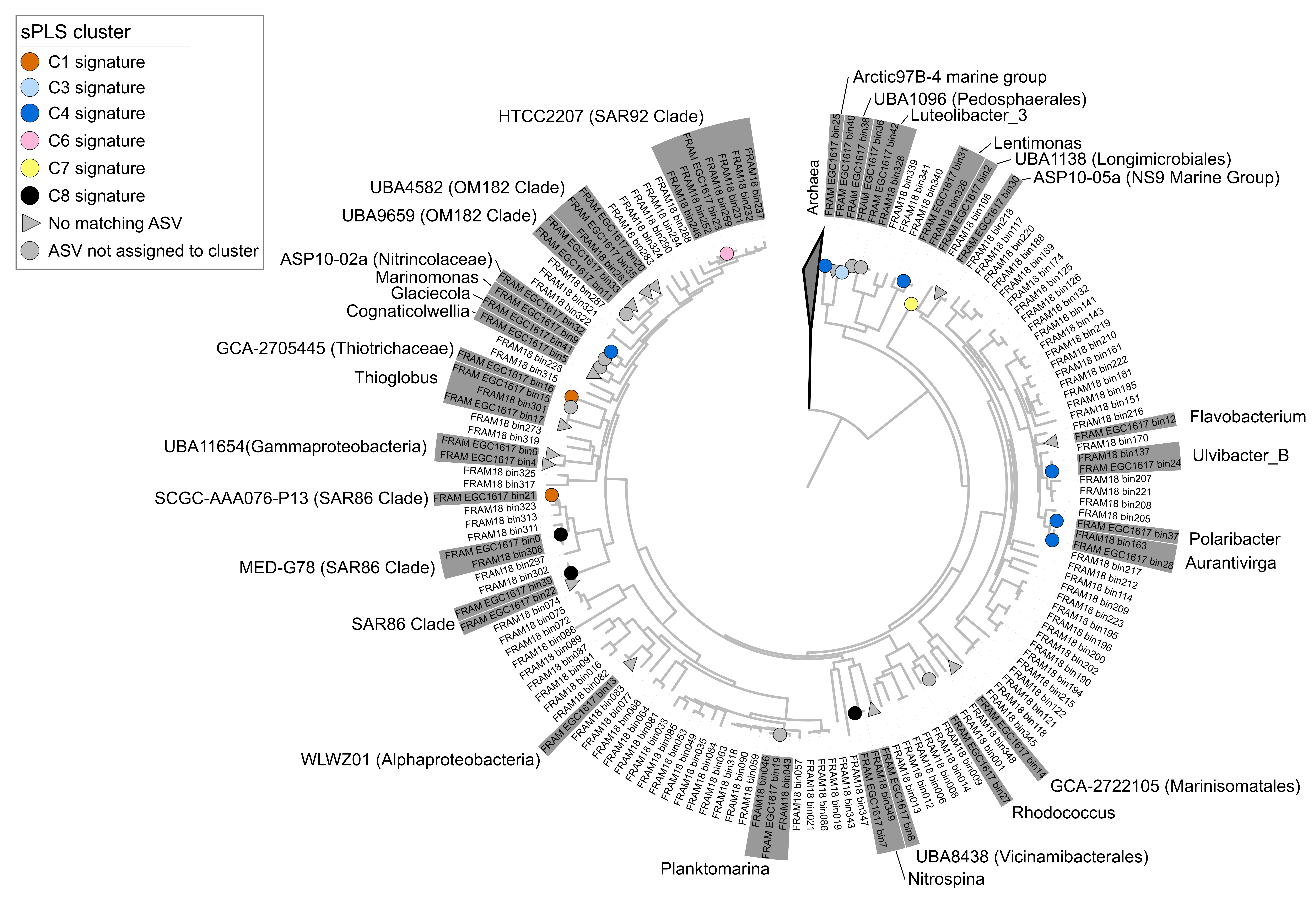

Supplement: Supplementary file 4 — Supplementary Figure S4 [file 41396_2023_1461_MOESM4_ESM.png]

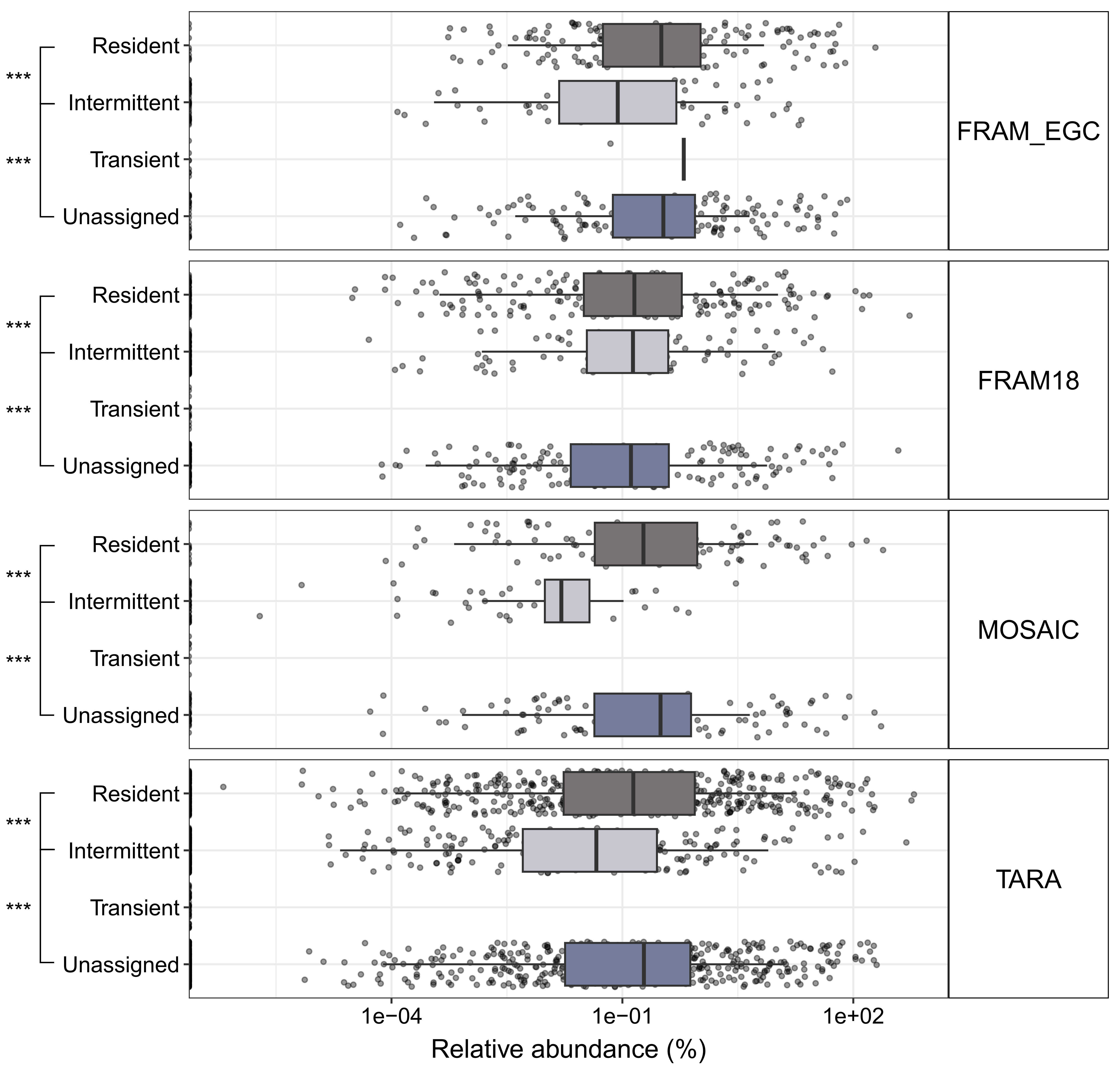

Supplement: Supplementary file 5 — Supplementary Figure S5 [file 41396_2023_1461_MOESM5_ESM.png]

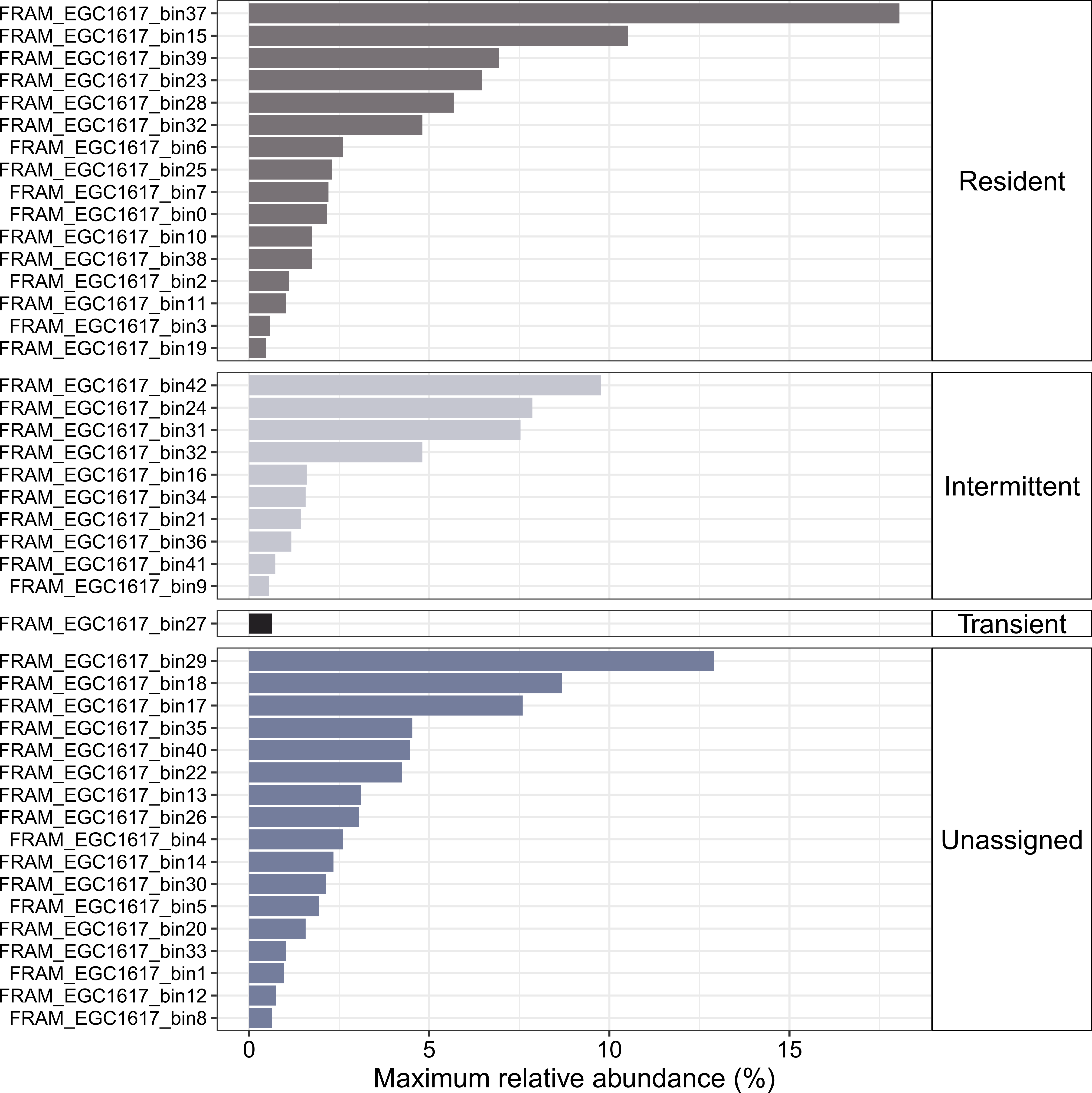

Supplement: Supplementary file 6 — Supplementary Figure S6 [file 41396_2023_1461_MOESM6_ESM.png]

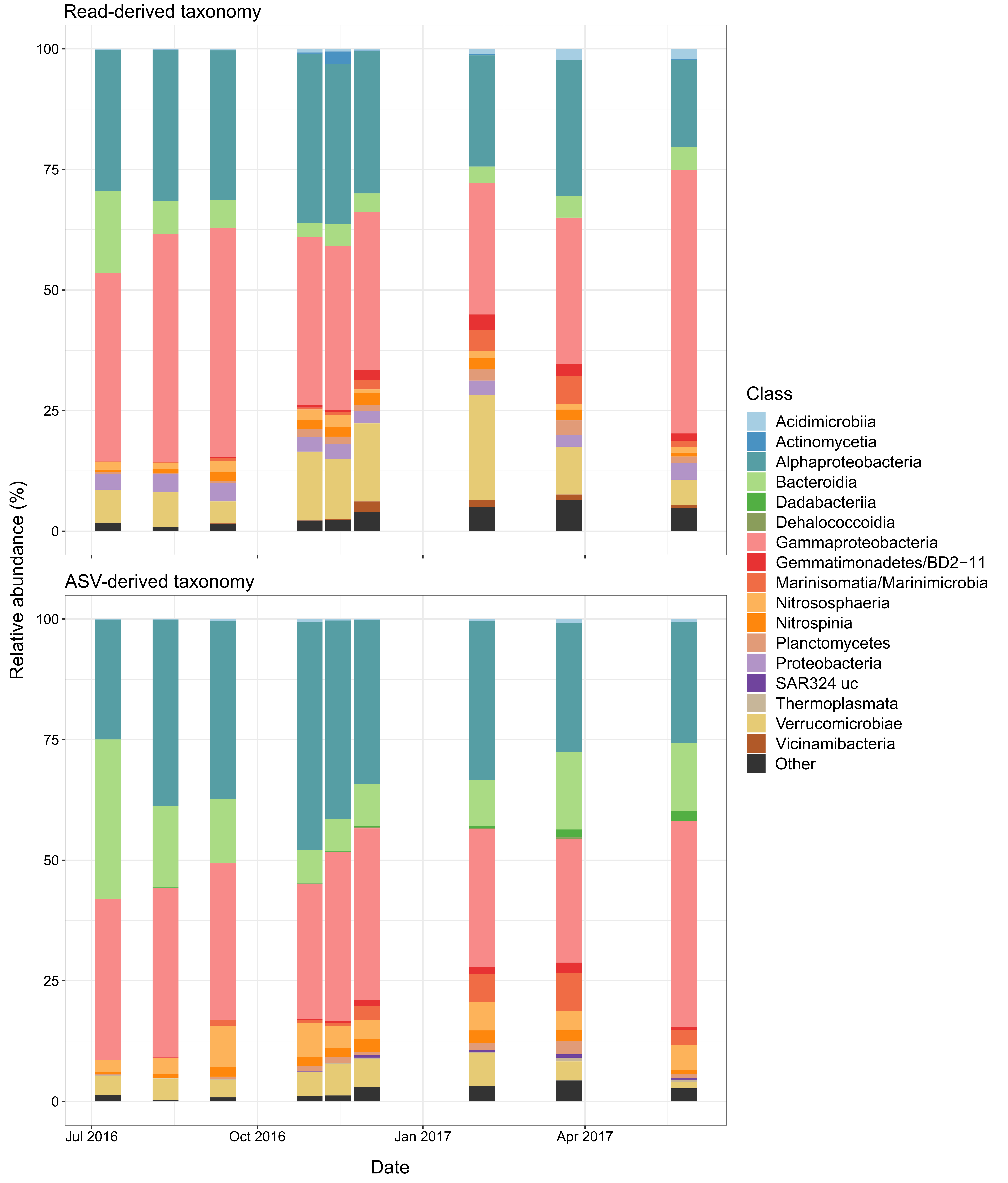

Supplement: Supplementary file 7 — Supplementary Figure S7 [file 41396_2023_1461_MOESM7_ESM.png]

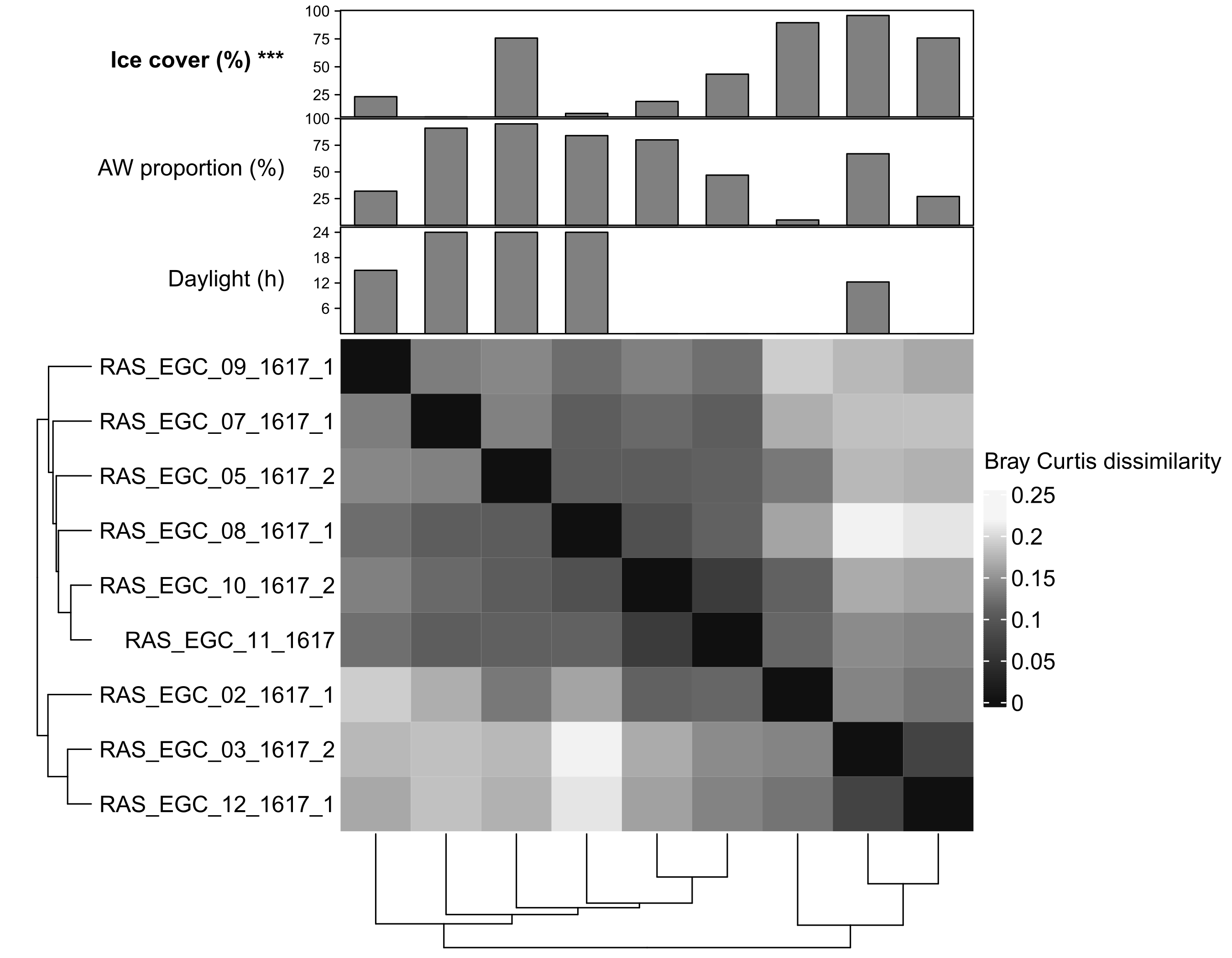

Supplement: Supplementary file 8 — Supplementary Figure S8 [file 41396_2023_1461_MOESM8_ESM.png]

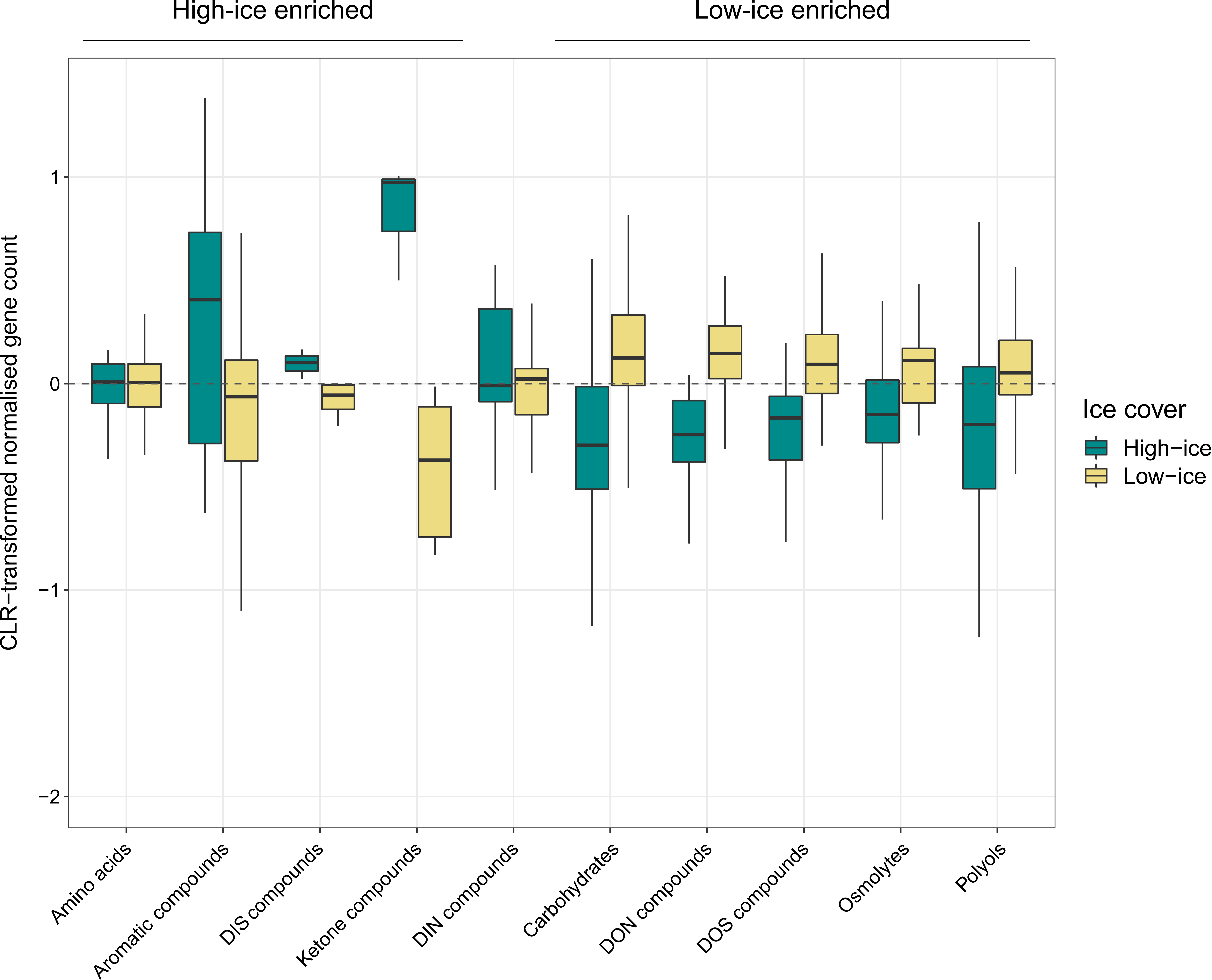

Supplement: Supplementary file 9 — Supplementary Figure S9 [file 41396_2023_1461_MOESM9_ESM.png]
