# Supplementary material for: Atlantic water influx and sea-ice cover drive taxonomic and functional shifts in Arctic marine bacterial communities
Source: ISME J. 2023 Jul 8;17(10):1612–25. doi: 10.1038/s41396-023-01461-6 (PMC10504371; doi:10.1038/s41396-023-01461-6)
Supplement: Supplementary file 10 — Supplementary Information [file 41396_2023_1461_MOESM10_ESM.pdf]

Supplementary Information: *Atlantic water influx and sea-ice cover drive taxonomic and functional shifts in Arctic marine bacterial communities*

SUPPLEMENTARY METHODS

Co-occurrence network analysis

The methodological description of the co-occurrence network analysis is provided in the main text. Here, we provide additional information on some of the key steps in the pipeline and why they were used. Co-occurrence networks were calculated using the normalized time-series abundance matrix from the collected samples. To determine co-occurrence between two ASVs, a Fourier transformation was conducted involving the decomposition of abundance information over time into wave frequencies or oscillation signals. This allows to analysis time-series on multiple levels, e.g. considering the amplitude of the signal as a proxy for abundance and phase shifts in the wave frequency to identify time lags or seasonal dynamics. Correlation analysis on these oscillation signals is a robust representation of co-occurrence within a time-series, as the correlation values project the time delay when ASVs have higher abundances compared to each other. For example, species with different amplitudes in their signals (different magnitudes in abundance) but that occur on the same day each year are considered as strongly co-occurring with a correlation of 1. Species that peak a single time per year with a time shift of ~45 days (0.25*pi) will show a correlation of 0.7. Using the oscillation signals allows to define the time of occurrence more precisely while being consistent with other analyses on the same time series.

SUPPLEMENTARY RESULTS

Co-occurrence networks

Networks were computed for both moorings separately in order to assess patterns in microbial population dynamics under Arctic- and Atlantic-dominated conditions. In the core-EGC network, Res-ASVs exhibited twofold more significant co-occurrences than Int-ASVs, averaging 29 and 13

respectively (Figure 3b). In contrast, Int-ASVs were more connected in the MIZ network. This pattern further illustrates the stability of the resident microbiome under Arctic-dominated conditions. In both networks, a distinct cluster of co-occurring, summerly-peaking (June-August) ASVs were observed. These included ASVs reaching the highest relative abundances, such as asv24-*Luteolibacter* (Int-ASV), asv6-*Polaribacter* (Res-ASV) and asv7-*Aurantivirga* (Res-ASV). The MIZ network exhibited additional strong seasonal structuring. No further co-occurrence patterns in relation to environmental conditions were observed in the core-EGC network (Supplementary Figure S3).

Distribution of signature populations across the Arctic Ocean

In order to validate our observations on signature population dynamics with environmental conditions and their assignment as resident, intermittent and transient, we assessed their distribution across the Arctic Ocean using the Tara Arctic prokaryote size-fraction dataset (Supplementary Figure S4). Signature population MAGs of the polar day clusters (C4 and C6) had, on average, highest relative abundances in upper euphotic zone samples (5-100 m depth). In particular, asv6-*Polaribacter* (C4) and asv16-SAR92 Clade (C6) showed average relative abundances of 2.7% and 2.1% respectively. At lower depths (>100 m), polar day signatures decreased and Arctic and polar night signature populations increased, i.e. as in C8 and C3, respectively. The most prominent were asv13-*Arenicellaceae* in cluster C3 and asv18-SAR86 Clade in cluster C8, with average relative abundances of 0.16 and 0.66% respectively. The Arctic Ocean sampling of Tara Oceans was conducted largely during polar day (May−October) above the continental shelf, with typically ice-free or low-ice conditions. As such, the higher prevalence of polar day signature populations (C4 and C6) in the upper water column is in agreement with their observed dynamics in the EGC. Water column stratification following sea-ice melt during polar day likely restricts Arctic and polar night signature populations to deeper waters. These populations are expected to increase in the upper water column in conjunction with deeper vertical

mixing in winter. Furthermore, “true” Arctic signature populations identified in our dataset are likely more prevalent in the Arctic Ocean basin, and are transported southward with water exiting the central Arctic through the EGC.

Ecological niches of signature populations

By combining temporal dynamics with functional gene predictions, we are able to make predictions on the ecological niches of signature populations within the context of the environmental conditions they represent. Signature populations from clusters C1 and C8 were characterized in detail in the main text. Here, we provide ecological descriptions for the remaining signature populations.

<u>Arctic signature populations.</u> The asv191 population was assigned to the Arctic97B-4 group (*Verrucomicrobiae*), for which no functional information is available to date. 16S rRNA gene-based studies have indicated elevated proportions of Arctic97B-4 in subsurface waters and a tight coupling with other deep water clades, such as SAR202 [1, 2]. An enrichment of Arctic97B-4-affiliated sequences was also identified in the small particle-attached fraction of Southern Ocean samples [3]. Functional annotations suggest a motile chemomixotroph with the capacity to fix carbon and assimilate sulfate. Comparable to other marine *Verrucomicrobia*, the asv191 population encoded a high number of CAZymes (23 genes) and sulfatases (84 genes). However, unlike other Verrucomicrobia, there was no clear specialisation on complex carbohydrates. The most numerous CAZyme gene families are associated with the degradation of animal-derived glycans, such as sialic acids – evidenced by numerous GH33 genes and a sialic acid transporter. In addition, we identified the capacity to synthesise riboflavin and biotin.

The cluster C7 representative, asv78, was assigned to two distinct classes, the BD2-11 terrestrial group (SILVA) and the *Gemmatimonadetes* (GTDB). This discrepancy reflects the relatively recent assignment and largely unresolved phylogeny of phylum *Gemmatimonadota*. Based on the few available cultured representatives and 16S rRNA-gene based studies, the

Gemmatimonadetes harbour aerobic/semi-aerobic chemoorganoheterotrophs inhabiting soil environments, but are also reported from freshwater and deep-sea sediments [4, 5]. Their presence in the upper marine water column is however rarely reported. The asv78 population encodes the capacity to use a wide range of organic substrates for growth, and also perform aerobic denitrification. However, the presence of periplasmic nitrate reductase (*nap*) and nitrite reductase (*nirK*) genes but absence of downstream genes required for further reduction to N_2 suggests an incomplete denitrification pathway. In addition, we identified genes to metabolise taurine, hypotaurine, D-amino acids, dicarboxylic acids and halogenated haloaliphatic compounds. The sources of these compounds in the marine environment vary, with taurine being attributed to phytoplankton and metazoa, D-amino acids to bacteria, and halogenated compounds to all forms of biota. With asv78's capacity to reduce nitrate, this would provide metabolic flexibility to prevail under low daylight conditions and high-ice cover.

The remaining Arctic signature populations were affiliated with *Nitrospina* (asv118) and the OM75 Clade (asv163), both of which exhibited similar dynamics and represented comparable proportions of the communities, reaching ~2% relative abundance. These populations harboured distinct functional features, relying on different substrates for growth but also performing key processes that contribute to the cycling of carbon, nitrogen and sulfur under high-ice cover. The asv163 population was assigned to the OM75 Clade in the SILVA database and the GCA-2722775 (within the *Nisaeaceae*) in the GTDB database. Functional gene annotations indicated a non-motile lifestyle with a photoheterotrophic metabolism, including a green-light proteorhodopsin and the capacity to use a diverse repertoire of organic substrates for C, N and S acquisition and energy generation. Of particular note was the extensive set of ABC transporters (53) compared to other Arctic signature populations (average of 17). Exogenous carbon substrates likely include osmolytes, one-carbon and aromatic compounds. The asv163 population encoded the C1 tetrahydrofolate oxidation pathway, similar to some members of the SAR11 clade [6], which also produces energy through ATP. The degradative pathways for the osmolytes

taurine, sarcosine and choline were encoded, plus partial metabolic pathways for aromatic compound metabolism. Organic compounds also represent a sulfur source for the asv163 population, with the degradation of sulfonates through 2-aminoacetaldehyde to sulfite. The sulfite could subsequently be converted to sulfate through the sulfite dehydrogenase gene (*soeABC*) and assimilated through 3'-phosphoadenylyl-sulfate. In addition to sulfonates, the asv163 population also harboured the capacity to utilise dimethylsulfide, dimethylsulfoxide and dimethylsulfone as sulfur and carbon source, with the production of formaldehyde being channeled through the tetrahydrofolate oxidation pathway. Organic nitrogen sources for the asv163 population are predicted to include amino acids and urea. Several branched-chain and L-amino acid transporters were unique to this population as well as the complete urease enzyme complex. The asv163 population thus harbours an extensive capacity to uptake and degrade diverse organic substrates, which would be advantageous under high-ice conditions with limited input of fresh organic matter.

The asv118 population, assigned to genus *Nitrospina* in SILVA and SCGCAAA288-L16 in GTDB, is a motile chemolithotroph, in line with descriptions from previous studies [7, 8]. *Nitrospina* belong to the *Nitrospinota* phylum that comprises the most abundant nitrite-oxidising bacteria (NOB) in the oceans. Identified from surface to deep waters and from oxygenated to oxygen minimum zones, *Nitrospinota* are essential for the marine nitrogen cycle, with microbial nitrite oxidation reported to be the most significant biological pathway for nitrate production in the oceans [9]. However, the essential nitrite reductase, *nirK* gene, was not identified in the asv118 population, which may reflect the lower completeness of the MAG. The presence of a nitrite:ferredoxin reductase, *nirA*, indicates a capacity to convert nitrite to ammonia in an assimilation process and reflects previous obvious in other nitrite oxidizing bacteria among *Nitrospira* [10]. Additional routes for nitrogen acquisition included ammonium uptake and a capacity to use urea through *ureABC*. Furthermore, asv118 encodes a green-light proteorhodopsin, suggesting supplemental energy generation through light − a feature not

previously reported for *Nitrospina* members. Although the key nitrite oxidation machinery was lacking, we hypothesise that this process would still formulate a key part of energy generation in this organism, due to its highly conserved nature across nitrite-oxidising bacteria. Recently, hydrogen oxidation was reported for nitrite-oxidising bacteria, however no such genes were identified in the asv118 representative MAG.

Atlantic signature populations. Correspondent to its abundance peak during polar day, the asv45 (*Thiotrichaceae*) population is predicted to utilise phytoplankton-derived organic substrates, including methanethiol and C1 compounds. Methanethiol is the product of dimethylsulfoniopropionate (DMSP) demthylation [11], an osmoprotectant produced by phytoplankton. DMSP concentrations in the Arctic Ocean show spatial variation and are influenced by water mass and sea-ice, with highest concentrations in the western Eurasian Arctic influenced by Atlantic water inflow [12]. The concentration of DMSP in these regions is tightly coupled to chlorophyll *a* [12, 13]. As such, methanethiol is likely more available in Atlantic waters under polar day conditions and could be a valuable substrate for the asv45 population. The oxidation of methanethiol through methanethiol oxidase (MTO) results in the production of formaldehyde and hydrogen sulfide. In the asv45 population, we identified the MTO gene along with a complete tetrahydromethanopterin (H4-MPT)-dependent oxidation pathway and sulfide oxidation machinery (*dsrAB* and *soeABC*). Combined, this genetic repertoire would allow the asv45 population to use methanethiol as a carbon, sulfur and energy source.

A similar metabolism has been reported for members of the *Rhodobacteraceae,* which are capable of degrading methanethiol and subsequently oxidising the hydrogen sulfide for energy generation [14]. The H4-MPT-dependent formaldehyde oxidation pathway has been traditionally affiliated with methanogenic bacteria but was also demonstrated in methylo- and methanotrophic members of the Alpha- and *Gammaproteobacteria* [15]. To our knowledge, this is the first such description in a sulfur-oxidizing member of the *Thiotrichaceae* family. Although experimental

evidence is needed to consolidate these findings, we confirmed the presence of the above-described pathways in each of the species-representative genomes from the assigned GTDB genus (GCA-2705445). The GCA-2705445 genus contains several representatives that are classified as *Thiothrix* in NCBI. The distinct metabolic features described above may represent unique characteristics and, in line with the GTDB classification, suggests that GCA-2705445 species are distinct from other *Thiothrix*.

The OM182 population differed from the other AW-associated signatures by showing daylight-independent dynamics. Functional gene annotations indicated a motile lifestyle with the capacity to oxidise sulfur and carbon monoxide (CO) as well as to degrade taurine and methylamine, thus representing an aerobic, sulfur-oxidising methylotroph. Furthermore, the presence of *sox*, polysulfide reductase (*pshAB*) and flavocytochrome c-sulfide dehydrogenase (*fccAB*) genes indicates the capacity to store and use elemental sulfur. The diverse metabolic capacities of the asv130 population may explain the observed dynamics over the time-series, with a capacity to switch nutrient and energy sources under different conditions. For example, under high daylight conditions, CO oxidation combined with the utilisation of organic compounds presumably provides sufficient energy and nutrients for growth. CO production in the oceans is linked to the photolysis of coloured dissolved organic matter and direct production by phytoplankton [16, 17], and thus concentrations would be elevated during polar day and in periods of high productivity. Under such conditions, the capacity to use taurine and methylamine − compounds related to phytoplankton production and organic matter degradation respectively − would provide further access to carbon, nitrogen and sulfur. CO oxidation as a supplemental energy source has been previously evidenced in some marine *Gammaproteobacteria* [18], however the dominant organisms performing such processes are typically affiliated with *Rhodobacteraceae*. In general, only few heterotrophic populations inhabiting the upper water column have been linked to sulfur and CO oxidation.

The Atlantic signature population asv157 was assigned to genus SCGC-AAA076-P13 within the SAR86 Clade. In contrast to the other Atlantic signature populations of cluster C1, this population peaked under polar night conditions in the MIZ, whereas no such pronounced peaks were observed in the core-EGC. SAR86 Clade members are heterotrophic organisms that are often also reported to harbour proteorhodopsin genes, being one of the most frequently observed groups in the upper marine water column. However, SAR86 contains a large phylogenetic diversity and at higher resolutions, distinct biogeographic patterns have been described [19, 20]. Due to the low completeness of the asv157 population representative MAG (56%), it is challenging to predict a potential ecological role. Similarly to the SAR86 Clade Arctic signature population (asv18), the asv157 population had a reduced capacity to degrade carbohydrates, with only a potential for β-1,3-glucan cleavage (2x GH17, 2x GH3), compared to proteins (14 peptidases) and also contained a proteorhodopsin, although here it was a blue-light adapted rhodopsin that is typical of surface water inhabiting microbes. As typical of SAR86 Clade members, this population is predicted to be heterotrophic, based on the presence of genes involved in glycolysis and the pentose phosphate pathway, but a lack of carbon fixation pathway genes. We additionally identified the genes involved in riboflavin biosynthesis (*ribABDEFH*).

<u>Polar day signature populations (clusters C3 and C6).</u> Seven signature populations were prevalent during polar day conditions; six assigned to the AW-associated cluster C3 and one to the PW-associated cluster C6. Despite the affiliations to different sPLS clusters, C3 signature populations also reached comparable relative abundance values at the core-EGC under polar day conditions, suggesting that water mass is less influential. In contrast, the asv16 (SAR92 Clade) PW-associated population did not reach comparably high relative abundances in MIZ samples, suggesting that this population is representative of polar day conditions only in Arctic water masses. Furthermore, the dynamics of the populations across the three polar day time periods in the core-EGC mooring appeared to be dependent on the magnitude of chlorophyll *a*

concentrations; this pattern indicates an intrinsic link to phytoplankton dynamics and suggests that a certain threshold of phytoplankton abundance must be reached to initiate a response in these populations. Each of the polar day signature populations are affiliated with taxa that are well-known to be responders to phytoplankton blooms in marine environments [21, 22]. At Helgoland Roads, a swift and recurrent succession of bacterial clades following phytoplankton blooms has been observed, with consecutive peaks of *Ulvibacter*, *Formosa, Reinekea, Polaribacter* and SAR92 [23]. Here, ephemeral peaks of some of these taxa exhibited a more coordinated and less successional pattern, however this may reflect the lower resolution of sampling. In general, asv6 (*Polaribacter*), asv7 (*Aurantivirga*), asv55 (*Ulvibacter*), asv71/88 (*Nitrincolaceae*) and asv16 (SAR92) exhibited rapid increases in relative abundance, peaking at the same time point followed by a decrease over a 4-week period. Although no chlorophyll *a* data is available from the MIZ, the dynamics in the core-EGC indicate that these patterns are a response to phytoplankton blooms. As such, we hypothesise that these populations are involved in the degradation of phytoplankton-derived organic matter, but each occupying distinct substrate-based niches, as has been observed at Helgoland Roads.

Aurantivirga and *Polaribacter* have been shown to harbour broad substrate utilisation capacities but also occupy distinct polysaccharide-based niches [24, 25]. In accordance with previous findings, both the asv6 (*Polaribacter*) and asv7 (*Aurantivirga*) representative MAGs harboured rich CAZyme gene repertoires and polysaccharide utilisation loci (PULs) for carbohydrate degradation. This consisted of 31 and 28 degradative CAZymes in asv6 and asv7, respectively, along with three distinct PULs in each. Two PULs identified in both of the populations (PUL1 containing GH16_3, GH17 and GH149, PUL2 containing GH16_3 and GH30_1) are predicted to target the diatom storage polysaccharide laminarin. The third PUL in the asv7 population is predicted to target α-glucans (via GH13, GH13_31 and maltose transporter). In the asv6 population, the third PUL (GH10, GH113, several sulfatases and D-xylose transporter) presumably targets sulphated xylose-containing polysaccharides. The gene synteny and

structures of the PULs agree with those previously described for *Polaribacter* and *Aurantivirga* representatives from Helgoland Roads [24]. Further comparisons revealed species-level differences in the dominant populations identified in our dataset and that of Helgoland Roads. For example, the asv6-*Polaribacter* population MAG shares 94.9% amino acid identity with 20120426_Bin_74_1 from Helgoland Roads (PRJEB28156), which was described as being present only in particular seasons and years but not related to *Polaribacter* species dominating during spring phytoplankton blooms. The above-described metabolic capacity, combined with ephemeral but pronounced peaks during polar day / chlorophyll *a* peaks in the EGC, indicate the occupation of distinct substrate-based ecological niches for the asv6 and as7 populations. Furthermore, the comparable relative abundance values at both core-EGC and MIZ suggest that these populations are capable of proliferating under contrasting conditions, suggesting that substrate availability is the key factor defining their ecological niche.

Also three signature populations affiliated with the *Verrucomicrobiae* exhibited pronounced increases in relative abundance. These populations were affiliated with the BACL24 (*Lentimonas*) and UBA1315 (*Luteolibacter*) genera. However, the asv94 population (*Lentimonas*) peaked with the *Bacteroidia* and *Gammaproteobacteria* representatives at core-EGC but showed a delayed response in the MIZ. The asv24 and asv115 (*Luteolibacter*) populations typically peaked later. These variations likely reflect the occupation of different ecological niches. Members of the *Verrucomicrobiae* are well evidenced to respond to phytoplankton blooms and are considered to degrade more complex polysaccharides, particularly those that are heavily sulphated [26, 27]. In accordance with previous findings, the *Luteolibacter* representatives encoded a large number of degradative CAZymes (40 in asv115 and 31 in asv24) and a high sulfatase to CAZyme ratio (1:0.8 in asv115 and 1:0.7 in asv24). Further analysis of CAZyme genes revealed key distinctions between the *Luteolibacter* populations. The asv24 population encoded genes assigned to five CAZyme families targeting alpha-glucans/amylose (GH13_38, GH13_4, GH13_8, GH57 and GH77), compared to only one gene in the asv115 population

(GH13_38). In addition, only the asv115 population encoded two CAZymes that target rhamnogalacturonan (GH105 and GH106). These metabolic distinctions may contribute to the large difference in maximum relative abundances between these populations (6% in asv115 and 15% in asv24), pointing towards substrate-based niche partitioning. Interestingly, the *Verrucomicrobiae* reported as the most prominent responders to spring phytoplankton blooms at Helgoland Roads are not from *Luteolibacter*, but affiliated with different genera of the *Akkermansiaceae* family or with *Lentimonas* of family *Puniceicoccaceae* [27]. In contrast, the *Lentimonas* population identified here (asv94) reached much lower relative abundances than the *Luteolibacter* populations. This further illustrates differences in the microbial populations that respond to phytoplankton blooms in different ecosystems.

In difference to the above-described polar day-associated representatives, the asv71/88 population harboured distinct metabolic features, including a capacity for methylotrophy and a rich genetic repertoire for sulfur metabolism. Classified as *Nitrincolaceae* in the SILVA database and ASP10-02a in GTDB, we characterize the asv71/88 population as a motile chemoheterotroph. Methylotrophic metabolism was evidenced by genes involved in trimethylamine utilisation (*tmm, dmd-tmd, mgsABC* and *mbdAB*), with the produced formaldehyde likely being converting to CO_2 through formate (*fdoG* and *fdwB*) and the ammonium being used as a nitrogen source. The asv71/88 population encoded a large number of genes involved in sulfur metabolism, including the ability to use organic sulfur compounds (methanesulfonate and sulfopyruvate) along with the complete thiosulfate oxidation machinery (*soxABCDXYZ*) and a sulfite dehydrogenase (*soeABC*). Methanesulfonate (MSA) is one of the main products of dimethylsulfide oxidation, and thus is likely present at higher concentrations during polar day and phytoplankton blooms. The oxidation of MSA through a MSA monooxygenase, encoded in the asv71/88 population, results in the production of formaldehyde and sulfite, which can be further oxidised through energy-generating reactions to CO_2 and sulfate. The asv71/88 population also harboured 26 ABC transporters mediating the uptake of amino acids, monosaccharides, polyols

and urea, as well as the potential to degrade toluene. Furthermore, we identified the genes for a mannose-sensitive haemagglutinin-like pilus (*mshCDGIJKLOP*), which has been shown to promote attachment of bacterial cells to algae [28]. Therefore, the ability for motility and attachment combined with a diverse metabolic capacity indicates a copiotrophic lifestyle that could involve close interactions with phytoplankton cells. Furthermore, encoded pathways for biotin, riboflavin, cobalamin and pantothenate synthesis suggest that vitamins may be provided to associated phytoplankton.

<u>Polar night signature populations (clusters C4).</u> Signature populations comprised asv13 (assigned to *Arenicellaceae* in SILVA and UBA11654 in GTDB) and asv8 (assigned to Arctic97B-4 in SILVA and UBA1096 in GTDB). The dynamics of these two populations were largely consistent, with highest relative abundances of ~4−5% during polar night in the MIZ. Insights into the metabolic capacity and potential ecological niches of these populations revealed some distinctions, however the asv13 representative MAG was of lower completeness (63%) reflected in the annotation of incomplete pathways. *Arenicellaceae* is a family of *Gammaproteobacteria* that has previously been reported in deep-sea sediments [29], responding to phytoplankton blooms in coastal seawater [30], and as indicator of eutrophication [31]. The limited ecological information on *Arenicellaceae* members indicates an organotrophic lifestyle. Due to the lower completeness of this MAG, we cannot provide clear predictions but will briefly outline key metabolic features found. In general, the asv13 population could be characterized as non-motile, with the capacity to use C1 compounds along with indications of nitrate reduction (nitrate reductase, *narH*) and carbon fixation (incomplete rTCA cycle). C1 metabolism was indicated by the presence of a methanol dehydrogenase, formate dehydrogenase, methylenetetrahydrofolate dehydrogenase (*folD*) and the complete pathway for cofactor F420 biosynthesis. Presence of a *narH* nitrate reductase suggests a potential for nitrate reduction, however the other key subunits were missing. Two ammonium channels illustrated additional routes for nitrogen acquisition. Transport systems

included typical transporters that are widespread in marine bacteria, such as vitamin B12, magnesium, sialic acid and general biopolymer transport (*exbBD*). Furthermore, we identified a complete riboflavin biosynthesis pathway. As a result, the ecological role of the asv13 population during polar night remains largely speculative.

The asv8 population was assigned to Arctic97B-4 in SILVA and hence the same taxonomic group as the Arctic signature population (asv191). In contrast, GTDB places the asv8 and asv191 populations into distinct families. This phylogenetic difference is also supported by distinctions in lifestyle and metabolic capacities. In difference to the Arctic signature population, the asv8 population can be categorised as non-motile and harbouring an extensive genetic repertoire for organic compound metabolism, particularly carbohydrates. A total of 55 degradative CAZymes and 54 sulfatases were identified, indicating considerable carbohydrate degradation capacity, which includes substrates such as pectin/rhamnogalacturonan (PL1 x 2, GH165 x 2, GH140 and CBM67 x 2), β-glucuronyl-containing polysaccharides (GH88, CE15 x 2) and sialic acids (GH33 x 3). These polysaccharides might be residual compounds from the productive season, or originate from ice algae and/or terrestrial-derived DOM. The asv8 population also encoded the capacity to use glyoxylate and dicarboxylates, such as glycolate (via *glcDEF*) as carbon and energy sources. For the acquisition of nitrogen and sulfur, inorganic sources are likely also used, attributed to the complete sulfate assimilation pathway and a nitrate reductase gene (*napA*); although not all necessary subunits and genes for complete nitrate reduction were found. Another key metabolic feature, which likely proves advantageous during winter conditions when fresh organic substrates are scarce, was the ability to synthesise glycogen (via GBE1 and *glgACE*). Glycogen plays important roles in energy and carbon storage in some bacteria and has been shown to increase bacterial durability during starvation [32]. Although the intracellular hydrolysis of glycogen would not provide sufficient resources to explain observed dynamics during winter, it could certainly aid in persisting during unfavourable conditions. Hence, the asv8 population might rely on inorganic substrates and complex, recalcitrant carbohydrates in winter.

SUPPLEMENTARY REFERENCES

1. Pajares S. Unraveling the distribution patterns of bacterioplankton in a mesoscale cyclonic eddy confined to an oxygen-depleted basin. *Aquat Microb Ecol* 2021; 87:151–166.
2. Chun S-J, Cui Y, Baek SH, Ahn C-Y, Oh H-M. Seasonal succession of microbes in different size-fractions and their modular structures determined by both macro- and micro-environmental filtering in dynamic coastal waters. *Sci Total Environ* 2021; 784:147046.
3. Milici M, Vital M, Tomasch J, Badewien TH, Giebel H-A, Plumeier I, et al. Diversity and community composition of particle-associated and free-living bacteria in mesopelagic and bathypelagic Southern Ocean water masses: Evidence of dispersal limitation in the Bransfield Strait. *Limnol Oceanogr* 2017; 62:1080–1095.
4. DeBruyn JM, Nixon LT, Fawaz MN, Johnson AM, Radosevich M. Global biogeography and quantitative seasonal dynamics of Gemmatimonadetes in soil. *Appl Environ Microbiol* 2011; 77:6295–6300.
5. Mujakić I, Piwosz K, Koblížek M. Phylum Gemmatimonadota and its role in the environment. *Microorganisms* 2022; 10:151.
6. Sun J, Steindler L, Thrash JC, Halsey KH, Smith DP, Carter AE, et al. One carbon metabolism in SAR11 pelagic marine bacteria. *PLOS ONE* 2011; 6:e23973.
7. Spieck E, Keuter S, Wenzel T, Bock E, Ludwig W. Characterization of a new marine nitrite oxidizing bacterium, *Nitrospina watsonii* sp. nov., a member of the newly proposed phylum “Nitrospinae”. *Syst Appl Microbiol* 2014; 37:170–176.
8. Luecker S, Nowka B, Rattei T, Spieck E, Daims H. The genome of *Nitrospina gracilis* illuminates the metabolism and evolution of the major marine nitrite oxidizer. *Front Microbiol* 2013; 4:27.
9. Mueller AJ, Jung M-Y, Strachan CR, Herbold CW, Kirkegaard RH, Wagner M, et al. Genomic and kinetic analysis of novel *Nitrospinae* enriched by cell sorting. *ISME J* 2021; 15:732–745.

10. Koch H, Lücker S, Albertsen M, Kitzinger K, Herbold C, Spieck E, et al. Expanded metabolic versatility of ubiquitous nitrite-oxidizing bacteria from the genus *Nitrospira*. *Proc Natl Acad Sci* 2015; 112:11371–11376.

11. Kiene RP. Production of methanethiol from dimethylsulfoniopropionate in marine surface waters. *Mar Chem* 1996; 54:69–83.

12. Uhlig C, Damm E, Peeken I, Krumpen T, Rabe B, Korhonen M, et al. Sea ice and water mass influence dimethylsulfide concentrations in the central Arctic Ocean. *Front Earth Sci* 2019; 7.

13. Park K-T, Lee K, Yoon Y-J, Lee H-W, Kim H-C, Lee B-Y, et al. Linking atmospheric dimethyl sulfide and the Arctic Ocean spring bloom. *Geophys Res Lett* 2013; 40:155–160.

14. Reisch C, Moran M, Whitman W. Bacterial catabolism of dimethylsulfoniopropionate (DMSP). *Front Microbiol* 2011; 2:172.

15. Vorholt JA, Chistoserdova L, Stolyar SM, Thauer RK, Lidstrom ME. Distribution of tetrahydromethanopterin-dependent enzymes in methylotrophic bacteria and phylogeny of methenyl tetrahydromethanopterin cyclohydrolases. *J Bacteriol* 1999; 181:5750–5757.

16. Gros V, Peeken I, Bluhm K, Zöllner E, Sarda-Esteve R, Bonsang B, et al. Carbon monoxide emissions by phytoplankton: evidence from laboratory experiments. *Environ Chem* 2009; 6:369–379.

17. Zuo Y, Jones RD. Formation of carbon monoxide by photolysis of dissolved marine organic material and its significance in the carbon cycling of the oceans. *Naturwissenschaften* 1995; 82:472–474.

18. Tolli JD, Sievert SM, Taylor CD. Unexpected diversity of bacteria capable of carbon monoxide oxidation in a coastal marine environment, and contribution of the *Roseobacter*-associated clade to total CO oxidation. *Appl Environ Microbiol* 2006; 72:1966–1973.

19. Hoarfrost A, Nayfach S, Ladau J, Yooseph S, Arnosti C, Dupont CL, et al. Global ecotypes in the ubiquitous marine clade SAR86. *ISME J* 2020; 14:178–188.

20. Dupont CL, Rusch DB, Yooseph S, Lombardo M-J, Alexander Richter R, Valas R, et al. Genomic insights to SAR86, an abundant and uncultivated marine bacterial lineage. *ISME J* 2012; 6:1186–1199.

21. Teeling H, Fuchs BM, Becher D, Klockow C, Gardebrecht A, Bennke CM, et al. Substrate-controlled succession of marine bacterioplankton populations induced by a phytoplankton bloom. *Science* 2012; 336:608–611.

22. Francis B, Urich T, Mikolasch A, Teeling H, Amann R. North Sea spring bloom-associated Gammaproteobacteria fill diverse heterotrophic niches. *Environ Microbiome* 2021; 16:15.

23. Teeling H, Fuchs BM, Bennke CM, Krüger K, Chafee M, Kappelmann L, et al. Recurring patterns in bacterioplankton dynamics during coastal spring algae blooms. *eLife* 2016; 5:e11888.

24. Krüger K, Chafee M, Ben Francis T, Glavina del Rio T, Becher D, Schweder T, et al. In marine Bacteroidetes the bulk of glycan degradation during algae blooms is mediated by few clades using a restricted set of genes. *ISME J* 2019; 13:2800–2816.

25. Avcı B, Hahnke RL, Chafee M, Fischer T, Gruber-Vodicka H, Tegetmeyer HE, et al. Genomic and physiological analyses of 'Reinekea forsetii' reveal a versatile opportunistic lifestyle during spring algae blooms. *Environ Microbiol* 2017; 19: 209–1221.

26. Sichert A, Corzett CH, Schechter MS, Unfried F, Markert S, Becher D, et al. Verrucomicrobia use hundreds of enzymes to digest the algal polysaccharide fucoidan. *Nat Microbiol* 2020; 5:1026–1039.

27. Orellana LH, Francis TB, Ferraro M, Hehemann J-H, Fuchs BM, Amann RI. Verrucomicrobiota are specialist consumers of sulfated methyl pentoses during diatom blooms. *ISME J* 2021;1–12.

28. Dalisay DS, Webb JS, Scheffel A, Svenson C, James S, Holmström C, et al. A mannose-sensitive haemagglutinin (MSHA)-like pilus promotes attachment of *Pseudoalteromonas tunicata* cells to the surface of the green alga *Ulva australis*. *Microbiology*; 152:2875–2883.

29. Deng L, Bölsterli D, Kristensen E, Meile C, Su C-C, Bernasconi SM, et al. Macrofaunal control of microbial community structure in continental margin sediments. *Proc Natl Acad Sci* 2020; 117:15911–15922.
30. Choi DH, An SM, Yang EC, Lee H, Shim J, Jeong J, et al. Daily variation in the prokaryotic community during a spring bloom in shelf waters of the East China Sea. *FEMS Microbiol Ecol* 2018; 94:fiy134.
31. Kopprio GA, Cuong LH, Luyen ND, Duc TM, Ha TH, Huong LM, et al. Carrageenophyte-attached and planktonic bacterial communities in two distinct bays of Vietnam: Eutrophication indicators and insights on ice-ice disease. *Ecol Indic* 2021; 121:107067.
32. Wang L, Wise MJ. Glycogen with short average chain length enhances bacterial durability. *Naturwissenschaften* 2011; 98:719.
